# The gut microbiota modulate locomotion via vagus-dependent glucagon-like peptide-1 signaling

**DOI:** 10.1038/s41522-024-00477-w

**Published:** 2024-01-16

**Authors:** Tzu-Ting Lai, Yu-Hsuan Tsai, Chia-Wei Liou, Ching-Hsiang Fan, Yu-Tian Hou, Tzu-Hsuan Yao, Hsiao-Li Chuang, Wei-Li Wu

**Affiliations:** 1 Institute of Basic Medical Sciences, College of Medicine, National Cheng Kung University, 1 University Rd., Tainan, 70101 Taiwan; 2 Department of Physiology, College of Medicine, National Cheng Kung University, 1 University Rd., Tainan, 70101 Taiwan; 3 Department of Biomedical Engineering, College of Engineering, National Cheng Kung University, 1 University Rd., Tainan, 70101 Taiwan; 4 National Laboratory Animal Center, National Applied Research Laboratories, Taipei, 115202 Taiwan

**Keywords:** Microbiome, Symbiosis

## Abstract

Locomotor activity is an innate behavior that can be triggered by gut-motivated conditions, such as appetite and metabolic condition. Various nutrient-sensing receptors distributed in the vagal terminal in the gut are crucial for signal transduction from the gut to the brain. The levels of gut hormones are closely associated with the colonization status of the gut microbiota, suggesting a complicated interaction among gut bacteria, gut hormones, and the brain. However, the detailed mechanism underlying gut microbiota-mediated endocrine signaling in the modulation of locomotion is still unclear. Herein, we show that broad-spectrum antibiotic cocktail (ABX)-treated mice displayed hypolocomotion and elevated levels of the gut hormone glucagon-like peptide-1 (GLP-1). Blockade of the GLP-1 receptor and subdiaphragmatic vagal transmission rescued the deficient locomotor phenotype in ABX-treated mice. Activation of the GLP-1 receptor and vagal projecting brain regions led to hypolocomotion. Finally, selective antibiotic treatment dramatically increased serum GLP-1 levels and decreased locomotion. Colonizing *Lactobacillus reuteri* and *Bacteroides thetaiotaomicron* in microbiota-deficient mice suppressed GLP-1 levels and restored the hypolocomotor phenotype. Our findings identify a mechanism by which specific gut microbes mediate host motor behavior via the enteroendocrine and vagal-dependent neural pathways.

## Introduction

Locomotion is a motor behavior crucial for survival in the wild and is necessary for activities such as escaping from predators, hunting prey, foraging for food, and searching for habitat^1–3^. Abnormal locomotor behavior is one of the key symptoms or comorbidities observed in individuals with several neurological diseases and mental disorders, such as Parkinson’s disease, Huntington’s disease, depression, anxiety, and psychosis^4–7^. Locomotion can be fine-tuned by various extrinsic and intrinsic factors, such as light, appetite, and energy consumption^8–10^. In rodent research, the open-field test (OFT) stands as a gold standard behavioral assay employed to evaluate locomotor activity, intricately linked with a spectrum of multifactorial dimensions. These encompass anxiety levels, exploratory tendencies, spatial memory, stress responses, and intricate movement patterns^11,12^. Interestingly, the commensal microbiota in the gut has been shown to impact locomotor behavior in different animal models to various degrees^13–16^. To a lesser extent, altered locomotion was observed in the animals with a depleted microbiome, including germ-free (GF) animals or animals treated with an antibiotic cocktail (ABX). However, a consistent outcome regarding the influence of microbial depletion on locomotion has not been observed in the field, possibly due to the differences in animal models, strains, ages, and sexes^13–22^. Therefore, whether and how the gut microbiota impacts host locomotion is still unclear.

Appetite is an intrinsic factor that serves as a driver to motivate forage-related behavior^9,23,24^. Several gut hormones secreted by enterocytes are responsible for appetite control. For example, cholecystokinin (CCK), glucagon-like peptide-1 (GLP-1), and peptide YY (PYY) are anorexigenic gut hormones that inhibit appetite; ghrelin is an orexigenic gut hormone that promotes appetite. Appetite-related hormones are the intrinsic factors driving food foraging and energy absorption^25,26^. Systemically and centrally injected analogs of appetite-related gut hormones into animals has been shown to alter locomotor activity, suggesting that gut hormones play a role in animal motor behavior^27–29^.

The vagus nerve is one of the most promising neural connections bidirectionally innervating the gastrointestinal (GI) tract and the brain^30,31^. It is considered the main constituent of the parasympathetic nervous system and comprises 80% afferent fibers and 20% efferent fibers^32^. Sensory input from the gut can be transduced rapidly within milliseconds to the nodose ganglion, brainstem and ascending brain regions for information integration^31^. Strikingly, neurodegenerative disease has been shown to be derived from the gut and spread to the brain through the vagus nerve interconnection^33^. Several studies have reported that the expression of gut hormone receptors, including those for CCK, GLP-1, PYY, and ghrelin, is distributed in vagal afferent terminals^30,34,35^. Based on its anatomical and functional characteristics, the vagus nerve is an important mediator of the interconnection of the peripheral and central nervous systems.

Commensal microorganisms inhabiting the GI tract have been shown to mediate appetite and metabolic homeostasis^36^. Dysbiosis of the gut microbiota can induce metabolic syndromes, inflammation, and hormone dysregulation^37–40^. Strikingly, the levels of CCK, GLP-1, and PYY as well as locomotion^13–16^ were found to be altered when the microbiota was depleted^37,41^. We speculate that the gut microbiota modulates locomotor activity via a mechanism involving the modulation of gut hormones. Herein, we discovered that the vagus-dependent microbiota-driven GLP-1 signaling pathway is crucial for the regulation of locomotion, a basic behavior critical for survival. Moreover, we identified specific microbes involved in the regulation of GLP-1 levels and locomotion.

## Results

### The gut microbiota affects locomotion via GLP-1 signaling

**Fig. 1:**
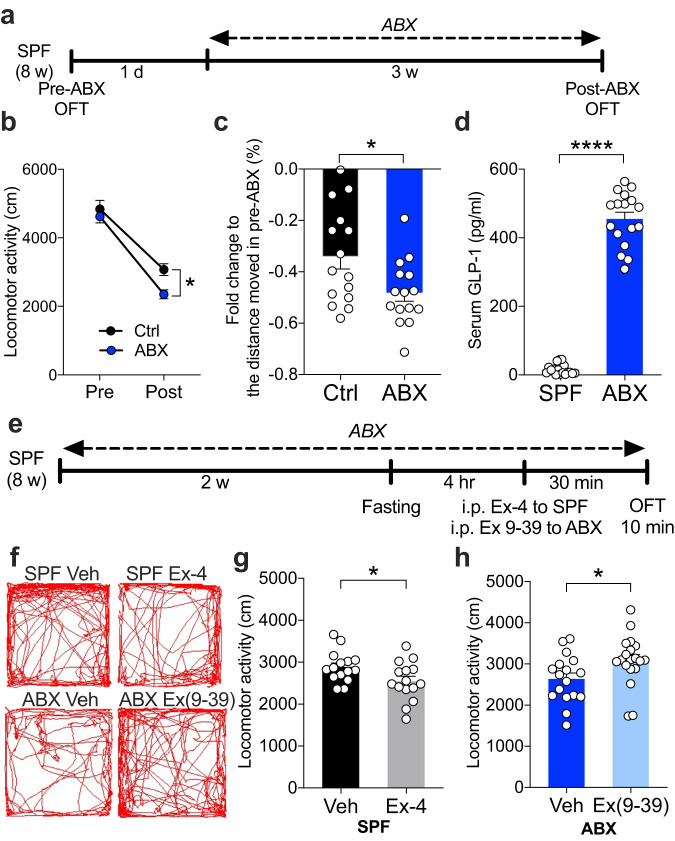
Depletion of gut microbiota alters locomotion via GLP-1 signaling in mice. **a** Timeline of the pre- and post-ABX treatment and OFT experiments. The mice were subjected to the OFT twice: before ABX treatment (pre-ABX) and three weeks after ABX treatment (post-ABX). **b** Locomotor activity was tested using the OFT paradigm in control and ABX mice pre- and post-ABX treatment (*n* = 15). **c** Fold change in the distance moved (locomotion) in pre-ABX mice. Hypolocomotion was observed after ABX treatment (*n* = 15). **d** Measurement of serum GLP-1 concentrations shows higher GLP-1 levels in the ABX-treated mice than in the SPF mice (*n* = 17). **e** Timeline of ABX treatment, fasting, Ex-4 or Ex(9-39) injection, and OFT experiments. The mice were fasted 4 hours prior to the injection of Ex-4 or Ex(9-39) and subjected to the OFT 30 min after injection. **f** Representative images of trajectories of OFT in SPF-Veh, SPF-Ex-4, ABX-Veh, and ABX-Ex(9-39) mice. **g** Locomotor activity was tested using the OFT paradigm in SPF-Veh and SPF-Ex-4 mice (*n* = 15). **h** Locomotor activity was tested using the OFT paradigm in ABX-Veh and ABX-Ex(9-39) mice. Locomotor activity in the whole arena (*n* = 17-19). Data represent the mean ± SEM. Data were analyzed by two-way repeated-measures ANOVA with Bonferroni’s multiple comparison post hoc test (**b**) and two-tailed unpaired *t-*test (**c**, **d**–**g**, **h**). *P < 0.05; ****P < 0.0001. SPF specific-pathogen-free, Ctrl control, ABX antibiotic cocktail, GLP-1 glucagon-like peptide-1, Veh vehicle, Ex-4 exenatide, Ex(9-39) exendin(9-39), OFT open-field test, w week, hr hour, min minute.

Animals displayed altered locomotion when their gut microbiome was depleted^13–16^, suggesting that locomotion is associated with the status of bacterial colonization, specific bacteria, and their metabolites^13–16^. To investigate the effect of a broad spectrum of ABX within mice at the adult stage, we performed the OFT before (pre) and after (post-) ABX treatment; control mice received vehicle (Fig. 1a). Both control and ABX mice displayed less locomotion during the second OFT (post) than during the first OFT (pre) due to the loss of novelty of the open-field chamber (Fig. 1b). Strikingly, the ABX-treated mice showed significantly less locomotion than the control mice during the second OFT (post) (Fig. 1b, c). We speculated that the reduction in locomotion in ABX mice was associated with the interoception of the gut, specifically appetite. Therefore, we tested locomotion under fasting conditions in specific-pathogen-free (SPF) or ABX mice. Interestingly, we observed that SPF mice exhibited more locomotion during the fasting state than the ABX mice (Supplementary Fig. 1a-c). The efficacy of microbiome depletion through ABX was validated through the quantification of bacterial load (Supplementary Fig. 1d). The observed reduction in locomotion during the OFT was unlikely to be attributed to the inclusion of sucrose in the ABX regimen. Notably, administering sucrose in the drinking water to SPF mice did not yield any significant alterations in their locomotor behavior (Supplementary Fig. 1e). ABX treatment exhibited no discernible impact on motor coordination, as evidenced by the results of the rotarod test (Supplementary Fig. 1f). Furthermore, the reduced locomotion in ABX mice was found only in the OFT but not in a novel cage (Supplementary Fig. 1g), suggesting that the hypolocomotion phenotype is context specific. These findings indicate that microbiome depletion via ABX during adulthood leads to a reduction in context-dependent locomotion.

Gut hormones have been suggested to be intrinsic factors driving motivation behaviors, including locomotion^25^. The secretion of enteroendocrine hormones (e.g., GLP-1, CCK, and PYY) is dysregulated in GF and ABX mice^37,41^. In addition, several studies have shown that administration of synthetic gut hormones in rodents affects their locomotor activity^27,29^. GLP-1 is a gut hormone regulated by the colonization status of the microbiota^37,41,42^. Similarly, we found that depletion of the gut microbiota by ABX dramatically increased GLP-1 levels in the serum and elevated GLP-1^+^ cells in the colon under basal conditions (Fig. 1d; Supplementary Fig. 2). Therefore, we next investigated whether GLP-1 modulates locomotion in mice. Both SPF and ABX mice were fasted before the test to control their fed state. SPF mice were intraperitoneally injected with the GLP-1 receptor (GLP-1R) agonist exenatide (Ex-4), and ABX mice were intraperitoneally injected with the GLP-1R antagonist exendin 9-39 (Ex(9-39)) (Fig. 1e). The SPF mice exhibited decreased locomotor activity in the Ex-4-injected group (Fig. 1f, g; Supplementary Fig. 1h). In contrast, intraperitoneal injection of Ex(9-39) successfully restored hypolocomotion in ABX mice (Fig. 1f, h; Supplementary Fig. 1i). To investigate the potential for an additive drug effect, we injected Ex(9-39) and Ex-4 reversibly to SPF and ABX mice, respectively. Remarkably, neither Ex-4 nor Ex(9-39) elicited any additional impact on locomotor behavior (Supplementary Fig. 1j–m).

CCK is another anorexigenic gut hormone with elevated gene expressions in the colon of ABX mice^37^. However, intraperitoneal injection of cholecystokinin-8 (CCK-8) did not affect locomotion in SPF mice (Supplementary Fig. 3a–f). Unexpectedly, ELISA measurements revealed a reduction in serum CCK levels in ABX mice (Supplementary Fig. 3g), contrasting with the findings reported for GF mice^43^. These results suggest the specificity of the microbiota and GLP-1 in the regulation of locomotion.

GLP-1 has been reported to impact emotion-related behavior, such as anxiety^44^. Therefore, it is speculated that GLP-1-mediated locomotion is driven by the altered anxiety response. We analyzed OFT behavior based on the distance traveled and time spent in the center and peripheral (thigmotaxis) zones. Interestingly, the decrease in locomotion in SPF mice injected with Ex-4 was more pronounced in the center zone of the OFT but not in the peripheral zone (Supplementary Fig. 1n). The increased locomotion in ABX mice injected with Ex(9-39) was more pronounced in the peripheral zone but not in the center zone (Supplementary Fig. 1o). We next analyzed the time spent in the center zone and the peripheral zone in the OFT and observed that Ex-4 produced anxiogenic effects in SPF mice, while Ex(9-39) had no effect on ABX mice (Supplementary Fig. 1p, q). A similar finding was observed in the elevated plus maze (EPM) test (Supplementary Fig. 1r, s), suggesting that the reduced locomotion in ABX mice was not due to anxiety. Moreover, the SPF mice injected with Ex-4 and ABX mice injected with Ex(9-39) did not show altered cognition in the novel object recognition test (Supplementary Fig. 1t, u). Altogether, these results demonstrate that the depletion of the microbiome increases the levels of GLP-1 and thus decreases locomotion.

### Vagal afferent neurons are necessary for hypolocomotion in ABX mice

**Fig. 2:**
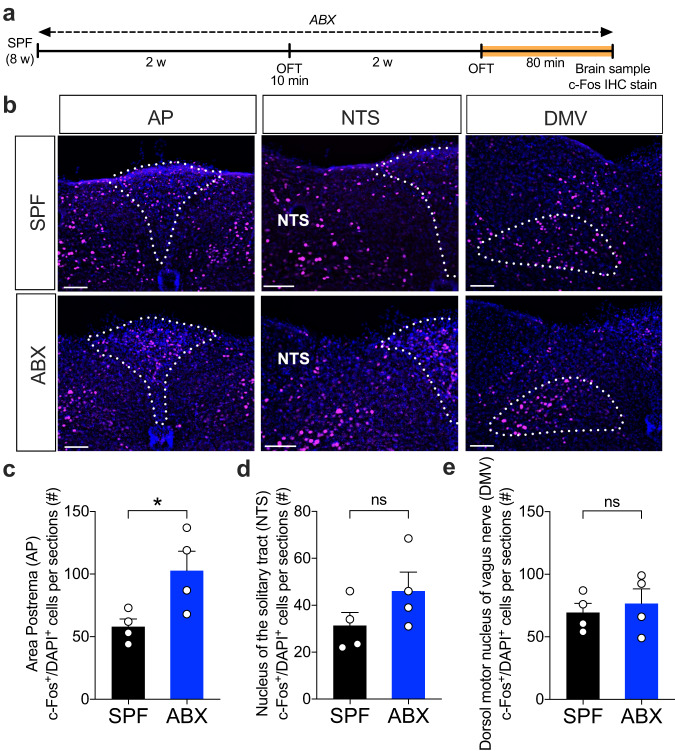
Higher numbers of c-Fos^+^ cells were detected in vagal ascending brain regions in ABX mice after OFT. **a** Timeline of sample harvesting. The brain was sampled 80 min after the OFT and subjected to immunohistochemistry. **b** Representative images of c-Fos staining in brain sections from SPF and ABX mice after OFT. Scale bar = 100 µm. **c**–**e** Quantification of c-Fos^+^ cells in the vagal ascending brain regions of SPF and ABX mice after OFT (*n* = 3–4). Data represent the mean ± SEM. Data were analyzed by two-tailed unpaired *t-*test (**c**–**e**). * *P* < 0.05; ns not significant. SPF specific-pathogen-free, ABX antibiotic cocktail, OFT open-field test, AP area postrema, NTS nucleus of the solitary tract, DMV dorsal motor nucleus of vagus nerve, w week, min minute.

To identify the neurons that are activated in ABX mice after OFT exposure, we analyzed the expression of c-Fos, a transcription factor associated with neuronal activity in a time dependent manner^45,46^, in the brains of SPF and ABX mice 80 min after OFT (Fig. 2a). We found an increase in the number of c-Fos^+^ cells in the area postrema (AP), a brain region belonging to the dorsal vagal complex (DVC), in ABX mice after the OFT (Fig. 2b, c). In addition, a trend toward an increase in the number of c-Fos^+^ cells was found in the nucleus of the solitary tract (NTS) (Fig. 2b, d). However, no difference was found in the dorsal motor nucleus of the vagus (DMV) between ABX and SPF mice, suggesting that vagal efferent neurons were not activated after the OFT (Fig. 2b, e). Concurrently, a similar elevation was observed in c-Fos^+^ cell expression within the NTS of SPF mice following Ex-4 administration (Supplementary Fig. 4a, b–d). However, the injection of Ex(9-39) into ABX mice did not result in the downregulation of c-Fos cells within the DVC area (Supplementary Fig. 4a, e–g). This finding prompted us to test whether the vagus nerve is a critical pathway for hypolocomotion in ABX mice.

**Fig. 3:**
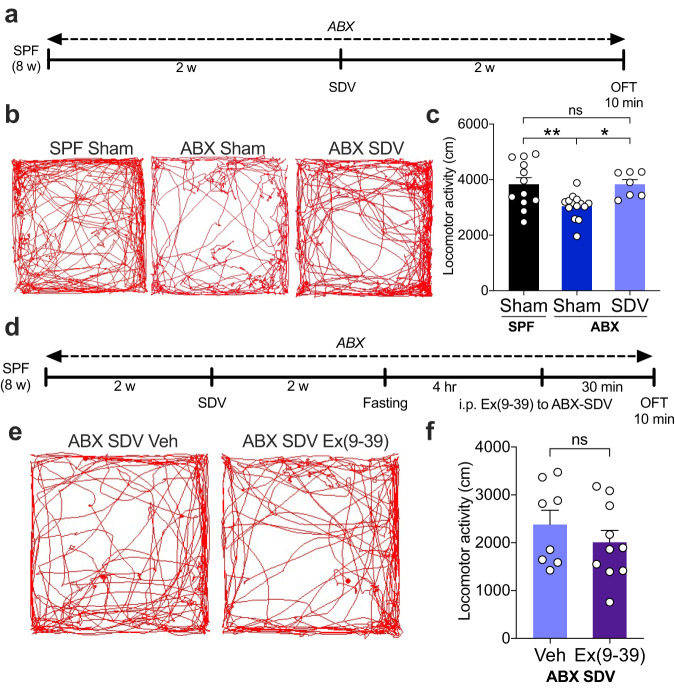
The subdiaphragmatic vagotomy (SDV) procedure restores hypolocomotion in ABX mice. **a** Timeline of the ABX treatment, SDV procedure, and OFT. The SDV procedure was executed two weeks after vehicle or ABX treatment. The mice were given two weeks to recover from SDV. **b** Representative images of trajectories of OFT in SPF-Sham, ABX-Sham, and ABX-SDV mice. **c** Locomotor activity was tested using the OFT paradigm in SPF-Sham, ABX-Sham, and ABX-SDV mice (*n* = 7–14). **d** Timeline of the ABX treatment, SDV procedure, Ex(9-39) injection, and OFT. The SDV procedure was executed two weeks after ABX treatment. The mice were given two weeks to recover from SDV. The mice were fasted 4 h prior to the injection of Ex(9-39) and tested in the OFT 30 min after injection. **e** Representative images of trajectories of OFT in vehicle- or Ex(9-39)-injected ABX-SDV mice. **f** Locomotor activity was tested using the OFT paradigm in vehicle- or Ex(9-39)-injected ABX-SDV mice (*n* = 8–10). Data represent the mean ± SEM. Data were analyzed by one-way ANOVA with Bonferroni’s multiple comparison post hoc test (**c**) and two-tailed unpaired *t-*test (**f**). *P < 0.05; **P < 0.01; ns not significant. SPF specific pathogen-free, ABX antibiotic cocktail, SDV subdiaphragmatic vagotomy, Veh vehicle, Ex(9-39) exendin(9-39), OFT open-field test, w week, hr hour, min minute.

Next, we performed the subdiaphragmatic vagotomy (SDV) procedure in ABX mice (Supplementary Fig. 5a, b) and tested their locomotion (Fig. 3a). The surgical completeness of the SDV was confirmed through multiple methods, encompassing resistance to CCK-8-induced anorexia (Supplementary Fig. 5c, d), observed stomach enlargement (Supplementary Fig. 5e), and retrograde neurotracing of the DMV using Fluorogold (Supplementary Fig. 5f–h)^47–49^. Strikingly, SDV rescued the hypolocomotion observed in ABX mice, while ABX-sham mice exhibited lower locomotion than SPF-sham mice (Fig. 3b, c). The locomotion in the 5-min bin was similar between SPF-sham and ABX-SDV, while ABX-sham showed an overall reduction in locomotion (Supplementary Fig. 6a). The diminishment of locomotor activity following SDV was not evident in SPF mice (Supplementary Fig. 6b). Moreover, SDV led to a reduction in the expression of c-Fos^+^ cells within the AP, NTS, and DMV regions in ABX mice after OFT (Supplementary Fig. 5i–l). No anxiety-like behavior (Supplementary Fig. 6c–e) or motor deficits (Supplementary Fig. 6f–i) were observed in ABX-sham or ABX-SDV mice. To validate whether vagal signaling in ABX mice is GLP-1 dependent, we injected ABX-SDV mice with Ex(9-39) intraperitoneally and tested their locomotion (Fig. 3d). Ex(9-39) did not produce any effect on locomotion (Fig. 3e, f; Supplementary Fig. 6j) or anxiety-like behaviors (Supplementary Fig. 6k–m) in ABX-SDV mice, unlike in ABX mice with an intact vagus nerve (Fig. 1f, h). Taken together, these results suggest that the vagus nerve is involved in a key GLP-1 signal transduction pathway that modulates locomotor activity in gut microbiota-deficient mice.

Transcranial focused ultrasound (FUS) is a noninvasive method used to modulate neural activity in the brain^50,51^. The DVC is a delicate brain area in the brainstem surrounded by brain regions vital for physiology^52,53^. The FUS strategy enabled us to target the mouse DVC in a noninvasive manner (Supplementary Fig. 7a, b). We validated c-Fos expression in SPF mice with a CCK-8-induced food intake test after FUS stimulation of the brainstem (Supplementary Fig. 7c). As expected, the number of c-Fos^+^ cells only increased in the AP of SPF-FUS mice unlike in SPF-sham mice (Supplementary Fig. 7c, d), but no difference was observed in other regions of the DVC (Supplementary Fig. 7c, e, f). Serum GLP-1 levels were unaltered by FUS in SPF mice (Supplementary Fig. 7g). SPF-FUS mice were then subjected to the OFT, and food intake was evaluated (Supplementary Fig. 7h-l). Interestingly, both the CCK-8- and saline-injected SPF-FUS groups showed less food intake than the SPF-sham mice (Supplementary Fig. 7h, i), which suggested a chronic anorexigenic effect produced by brainstem FUS stimulation. Strikingly, locomotion was decreased in SPF-FUS mice (Supplementary Fig. 7j–l). No overt motor dysfunction was observed after FUS stimulation in mice (Supplementary Fig. 7m–p). These results suggested that FUS stimulation in the brainstem can recapitulate the hypolocomotion phenotype similar to ABX mice.

### Selective antibiotic treatment alters GLP-1 levels and the gut microbiome

**Fig. 4:**
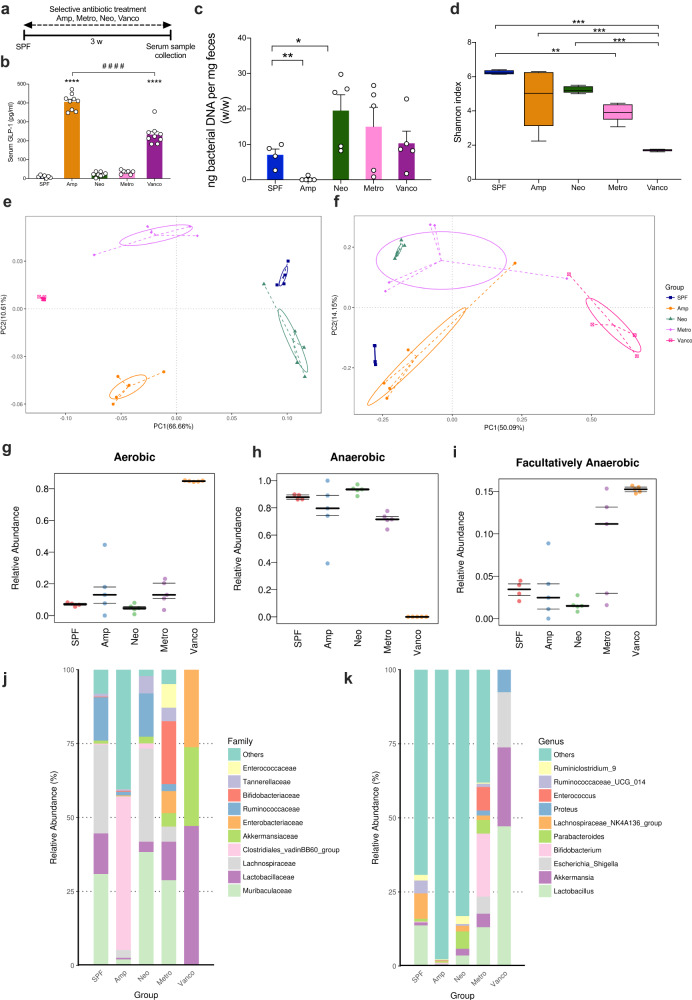
Selective antibiotic treatment alters GLP-1 levels and the gut microbiome in mice. **a** Timeline of the selective antibiotic treatment and serum and feces collection. The SPF mice were treated with selective antibiotics for three weeks before sample collection. **b** Measurement of serum GLP-1 concentrations shows elevation in the Amp-treated and Vanco-treated groups (*n* = 7–9). **c** Fecal bacterial DNA quantification was detected by Femto DNA quantification methods in SPF, Amp, Neo, Metro, Vanco mice (*n* = 5). **d** Alpha diversity analysis in Simpson index in SPF, Amp-, Neo-, Metro- and Vanco-treated mice (*n* = 4–5). **e** PCA plots based on weighted UniFrac distances among the gut microbiome of SPF, Amp-, Neo-, Metro- and Vanco-treated mice (*n* = 4–5). **f** PCA plots based on unweighted UniFrac distances among the gut microbiome of SPF, Amp-, Neo-, Metro- and Vanco-treated mice (*n* = 4–5). **g** BugBase prediction of aerobic bacterial communities in SPF, Amp-, Neo-, Metro- and Vanco-treated groups (*n* = 4–5). **h** BugBase prediction of anaerobic bacterial communities in SPF, Amp-, Neo-, Metro- and Vanco-treated groups (*n* = 4–5). **i** BugBase prediction of facultatively anaerobic bacterial communities in SPF, Amp-, Neo-, Metro- and Vanco-treated groups (*n* = 4–5). **j** Relative abundance in SPF, Amp-, Neo-, Metro- and Vanco-treated groups in top 10 family levels (*n* = 4–5). **k** Relative abundance in SPF, Amp-, Neo-, Metro- and Vanco-treated groups in top 10 genus level (*n* = 4–5). Data represent mean ± SEM (**b**, **c**) box plots show median (centerline) and interquartile range (IQR) with 1.5 times IQR of the upper and lower quartiles (**d**) horizontal lines in BugBase prediction represent the first quartile, the median, and the third quartile (**g**–**i**). Data were analyzed by one-way ANOVA with Bonferroni’s multiple comparison post hoc test (**b**) two-tailed unpaired *t-*test (**c**) Kruskal–Wallis test with LSD post hoc test (**d**). * *P* < 0.05; **P < 0.01; ***P < 0.001; ****P < 0.0001. Panel **b**: ****P < 0.0001 versus SPF, ^####^*P* < 0.0001 versus Amp. SPF specific pathogen-free, Amp ampicillin, Neo neomycin, Metro metronidazole, Vanco vancomycin, PCA principal coordinate analysis, w week.

The complex gut commensal microbiota plays a crucial role in modulating the levels of gut hormones^37,41^. Treatment with broad-spectrum ABX, including ampicillin, metronidazole, neomycin, and vancomycin, dramatically increased GLP-1 levels in the serum (Fig. 1d). To identify the key bacteria mediating GLP-1 levels in the host, mice were treated with a single antibiotic administered in their drinking water for three weeks (Fig. 4a). Strikingly, the levels of GLP-1 were significantly higher only in the ampicillin- and vancomycin-treated mice than in SPF mice but not in metronidazole- and neomycin-treated mice (Fig. 4b). The selective antibiotic treatment allowed us to profile the microbiome composition and to evaluate the association between the microbiome and GLP-1.

We collected fecal samples, extracted bacterial DNA, quantified the absolute levels of bacterial DNA, and sequenced the V3-V4 regions of 16S ribosomal RNA (16S rRNA). Our analysis revealed significantly diminished levels of bacterial DNA in ampicillin-treated mice, indicating the effective suppression of intestinal bacterial growth by ampicillin treatment (Fig. 4c). Regarding the alpha diversity, the difference between ampicillin-treated mice and SPF mice was less pronounced than the difference between vancomycin-treated mice and SPF mice (Fig. 4d; Supplementary Fig. 8a–c). Regarding beta diversity, principal coordinates analysis (PCoA) and nonmetric multidimensional scaling (NMDS) indicated that selective antibiotic treatment led to different microbiomes in the gut. Four different classes of antibiotics distinguishably shaped the mouse microbiome, as shown by weighted (Fig. 4e) and unweighted (Fig. 4f) UniFrac in PCoA and in the NMDS plot (Supplementary Fig. 8d). The relative abundance of fecal bacteria determined using the BugBase prediction of bacterial phenotypes (Fig. 4g–i) at the top 10 family (Fig. 4j) and genus (Fig. 4k) levels demonstrated dysbiosis produced by individual antibiotic treatment, especially vancomycin treatment.

**Fig. 5:**
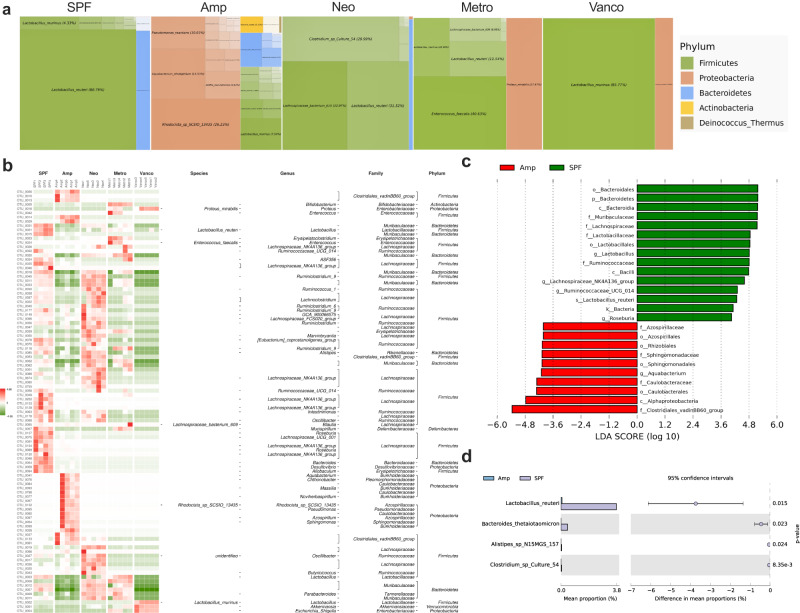
Relative abundance of microbes of the microbiota in selective antibiotic-treated mice. **a** Treemap showing the enriched bacterial species in SPF, Amp-, Neo-, Metro- and Vanco-treated mice (*n* = 4–5). **b** Z score heatmap of the bacterial taxa in SPF, Amp-, Neo-, Metro- and Vanco-treated mice (*n* = 4–5). **c** LEfSe comparison of the bacterial taxa between SPF and Amp-treated mice (*n* = 4–5). **d** STAMP comparison between the SPF and Amp-treated groups at the species level (*n* = 4–5). Data were analyzed by nonparametric factorial Kruskal‒Wallis sum-rank test (**c**) Welch’s *t-*test (**d**). SPF specific pathogen-free, Amp ampicillin, Neo neomycin, Metro metronidazole, Vanco vancomycin, LEfSe linear discriminant analysis effect size, STAMP statistical analysis of metagenomic profiles.

To identify the features of the microbial dysbiosis driven by the selective antibiotic, the Treemap (Fig. 5a) and Z score heatmap (Fig. 5b) via operational taxonomic unit (OTU) analysis of 16S rRNA were generated, and the results showed the relative abundance of fecal bacteria at the species level. We compared the bacterial taxa in animals treated with ampicillin (Fig. 5c) and with vancomycin (Supplementary Fig. 9a), and the two antibiotics upregulated GLP-1 levels (Fig. 4b) in SPF mice as revealed by linear discriminant analysis effect size (LEfSe) analysis. Moreover, the significant differences in special bacterial taxa were validated by Welch’s test by statistical analysis of metagenomic profiles (STAMP) (Fig. 5d; Supplementary Fig. 9b). The abundances of the commensal bacteria *L. reuteri, B. thetaiotaomicron*, *Alistipes_sp_N15MGS_157*, and *Clostridium_sp_Culture_54* were convergently found to be decreased in both ampicillin- (Fig. 5d) and vancomycin-treated mice (Supplementary Fig. 9b). Canonical correspondence analysis (CCA) revealed that the relative abundances of *L. reuteri* and *B. thetaiotaomicron* were negatively correlated with the levels of GLP-1 (Supplementary Fig. 9c). The functional prediction performed using phylogenetic investigation of communities by reconstruction of unobserved states (PICRUSt) and Kyoto encyclopedia of genes and genomes (KEGG) implicated that the depleted gut microbiome was associated with metabolic pathways, including those related to carbohydrates, butanoate, and amino acids (Supplementary Fig. 9d). Altogether, these results demonstrate that a selective antibiotic strategy enabled us to identify the specific bacterial taxa associated with GLP-1 levels.

### Specific microbes modulate GLP-1 levels and locomotion

Previous studies have shown that colonization by *B. thetaiotaomicron* in GF animals reduced the GLP-1^+^ cells in the intestine but did not alter the circulating GLP-1 levels^54,55^. Interestingly, the administration of *B. thetaiotaomicron* in ethanol-containing diet or *L. reuteri* in high-fructose diet restored the lowered GLP-1 levels systematically^56,57^, which is contradictory to our microbiome analysis. In addition, it was reported that *L. reuteri* possesses the capability to impact the host behavior through vagus nerve^58^. Based on these implications, we speculate that *L. reuteri* and *B. thetaiotaomicron*, the two bacteria candidates selected from our microbiome analysis, can modulate host GLP-1 levels and locomotion.

**Fig. 6:**
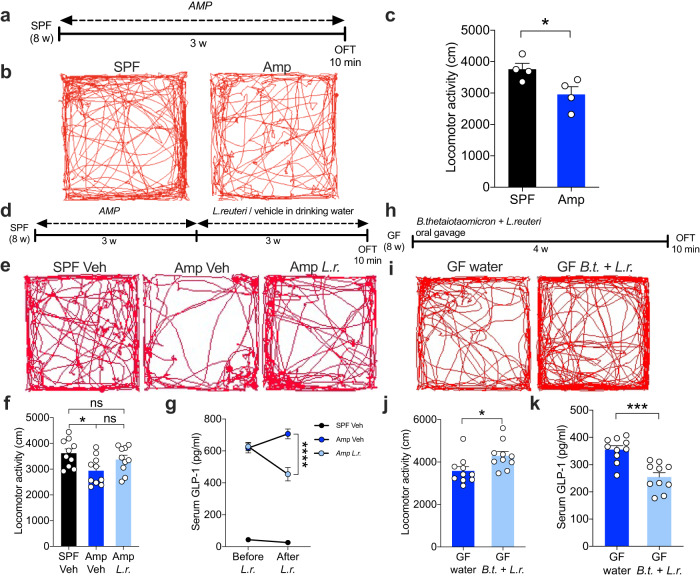
*L. reuteri* and *B. thetaiotaomicron* decrease GLP-1 levels and promote locomotion. **a** Timeline of Amp treatment and OFT. The OFT was performed three weeks after ampicillin treatment. **b** Representative images of trajectories of OFT in SPF and ampicillin-treated mice. **c** Locomotor activity was tested using the OFT paradigm in SPF and ampicillin-treated mice (*n* = 4). **d** Timeline of ampicillin treatment, *L. reuteri* colonization, and OFT. The drinking water was changed every two days for three weeks until the behavioral test. **e** Representative images of trajectories of OFT in SPF-Veh, Amp-Veh, and Amp-*L.r*. mice. **f** Locomotor activity was tested using the OFT paradigm in SPF-Veh, Amp-Veh, and Amp-*L.r*. mice (*n* = 10). **g** Measurement of serum GLP-1 concentrations before and after *L.reuteri* colonization in SPF-Veh, Amp-Veh, and Amp-*L.r*. mice (*n* = 10). **h** Timeline of bacterial colonization and OFT. GF mice received single oral gavage of *L. reuteri* and *B. thetaiotaomicron*, and the OF *T*-test was performed four weeks after colonization. **i** Representative images of trajectories of OFT in GF and GF-*B.t.+ L.r*. mice. **j** Locomotor activity was tested using the OFT paradigm in GF and GF-*B.t.+ L.r*. mice (*n* = 10). **k** Measurement of serum GLP-1 concentrations in GF, GF-*B.t.+L.r*. mice (*n* = 10). Data represent mean ± SEM. Data analyzed by two-tailed unpaired *t-*test (**c**–**j**, **k**) one-way ANOVA (**g**) and two-way ANOVA with repeated measures (**g**) with Bonferroni’s multiple comparison post hoc test. *P < 0.05; **P < 0.01; ***P < 0.001; ****P < 0.0001; ns no significant. SPF specific-pathogen-free, Amp ampicillin, Veh vehicle, *L.r*. *Lactobacillus reuteri,* *B.t*. *Bacteroides thetaiotaomicron,* GF germ-free, GLP-1 glucagon-like peptide-1, w week, min minute.

To evaluate whether *L. reuteri* and *B. thetaiotaomicron* are capable of suppressing GLP-1 levels and, most importantly, locomotion in an antibiotic-treated mouse model, we first tested locomotor activity in ampicillin-treated mice following the previously described procedure (Fig. 6a). Consistently, ampicillin-treated mice exhibited less locomotion than SPF mice (Fig. 6b, c). Next, we colonized ampicillin-treated mice with *L. reuteri* administered through drinking water for three weeks (Fig. 6d; Supplementary Fig. 10a, b). Strikingly, *L. reuteri*-colonized mice displayed similar levels of locomotion to the SPF vehicle mice, while ampicillin-treated mice displayed less locomotion than the SPF vehicle mice (Fig. 6e, f; Supplementary Fig. 10c). The serum GLP-1 levels were partially lower in *L. reuteri*-colonized mice than the ampicillin vehicle mice, as expected (Fig. 6g). *L. reuteri* was demonstrated to alter behavior through the oxytocin receptor in the brain^58^. To investigate whether *L. reuteri* normalized locomotion through the oxytocin receptor pathway, we administered the oxytocin receptor antagonist L371,257 intranasally and tested locomotion in *L. reuteri*-colonized mice. No difference was observed in locomotion between L371,257-treated *L. reuteri*-colonized mice and control mice (Supplementary Fig. 10d-g).

The influence of bacterial colonization in ampicillin-treated mice on locomotor activity was conspicuously absent in mice colonized with *B. thetaiotaomicron* (Supplementary Fig. 10h–l). To achieve this, *B. thetaiotaomicron* was administered via gavage to ampicillin-treated mice six times over the course of three weeks, followed by the assessment of locomotion and GLP-1 levels (Supplementary Fig. 10h). Notably, successful colonization by *B. thetaiotaomicron* was confirmed (Supplementary Fig. 10i), yet it did not elicit any alteration in the locomotor behavior of ampicillin-treated mice (Supplementary Fig. 10j, k). Intriguingly, a reduction in GLP-1 levels was observed in mice colonized with *B. thetaiotaomicron* (Supplementary Fig. 10l). These findings support the notion that the bacterial candidates *L. reuteri* and *B. thetaiotaomicron*, selected through antibiotic-based screening and microbiome sequencing, possess the capacity to modulate circulating GLP-1 levels. However, their effects on locomotor activity appear to be selective in nature.

Additionally, we co-colonized GF mice with *L. reuteri* and *B. thetaiotaomicron* and subjected them to the OFT (Fig. 6h; Supplementary Fig. 10m–q). Notably, GF mice exhibited a baseline increase in locomotor activity during the OFT (Supplementary Fig. 10m). Strikingly, GF mice co-colonized with *L. reuteri* and *B. thetaiotaomicron* displayed a notable augmentation in locomotor behavior (Fig. 6i, j; Supplementary Fig. 10q), coupled with reduced levels of GLP-1 (Fig. 6k) when compared to the GF control mice. Taken together, these results suggest that we identified specific microbes involved in the modulation of GLP-1 levels and locomotion.

## Discussion

**Fig. 7:**
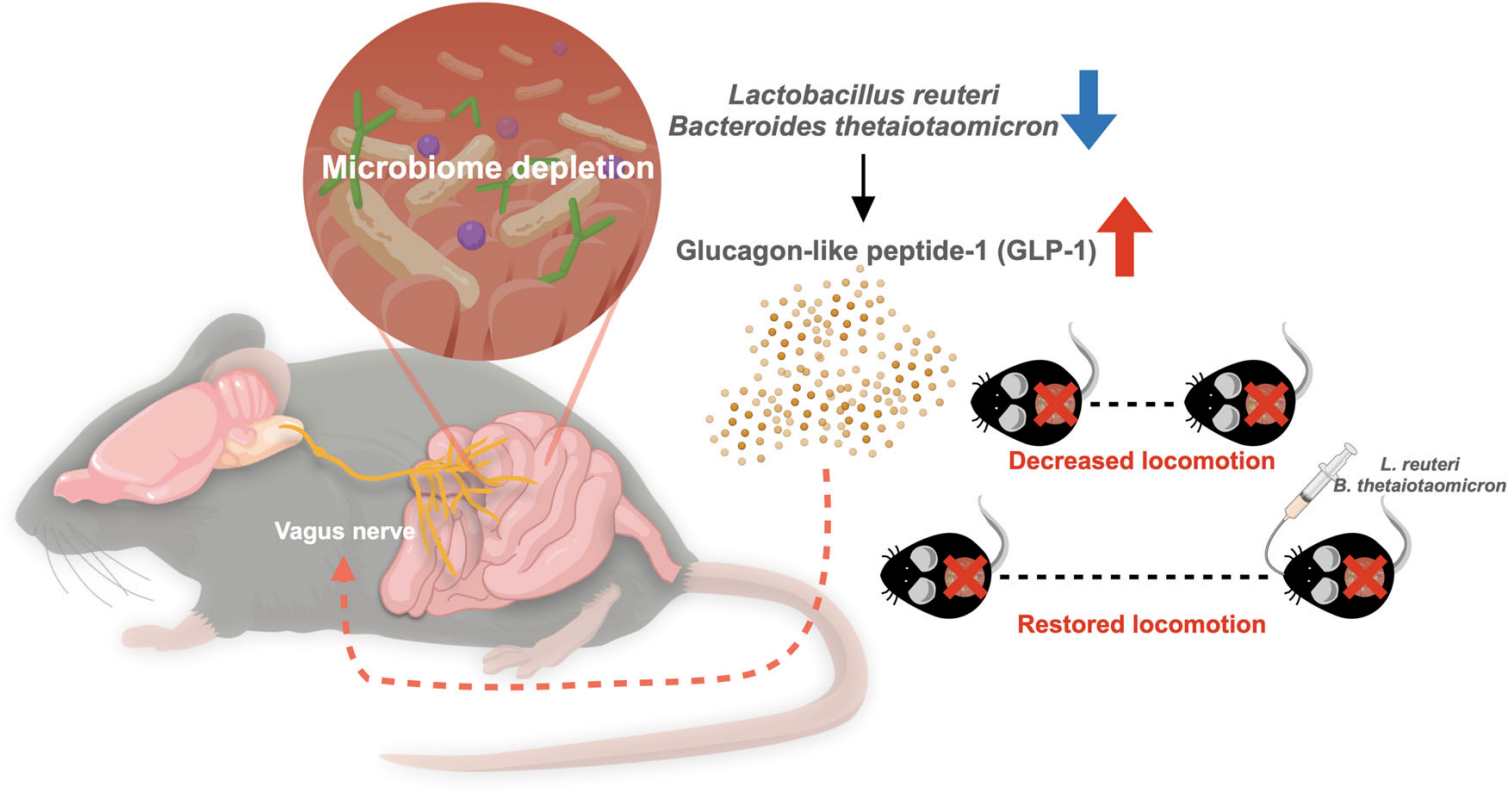
A schematic model of commensal microbiota mediate mouse movement by vagus nerve and circulating glucagon-like peptide-1.

This study demonstrated that depletion of the gut microbiome by administering ABX to adult mice increased GLP-1 levels in the circulation and induced hypolocomotion. Hypolocomotion in ABX mice (absence of microbiome) can be rescued by systemic antagonism of GLP-1R and incision of the subdiaphragmatic vagus nerve by SDV. Reciprocally, hypolocomotion can be systematically observed in SPF mice (presence of microbiome) subjected to agonism of GLP-1R and neural modulation of DVC by FUS. Ampicillin or vancomycin treatment selectively precluded gut microbes from suppressing GLP-1 levels in the host. Colonization by the identified bacteria, *L. reuteri* and *B. thetaiotaomicron*, in microbiota-deficient mice not only decreased GLP-1 levels but also restored locomotion. Taken together, these results suggest that vagus-dependent enteroendocrine signaling serves as a mediator of specific gut microorganisms to modulate locomotor behavior (Fig. 7). This finding provides a new concept of distal signaling in the gut-brain circuit for the regulation of locomotion.

Recent studies have reported that microbial colonization status is closely associated with locomotor behavior^13–16^. However, age, sex, genetic background, or model organisms might profoundly impact the outcome. For example, adult male GF mice with NMRI and a Balb/c background displayed hyperlocomotion in the OFT^14,15^. Another study reported that GF C57BL/6 J mice exhibited hypolocomotion at the age of 4 weeks but no change in locomotion at the age of 12 weeks^16^. Interestingly, a study conducted by the Petterson group revealed that C57BL/6 J mice displayed reduced locomotor activity in adulthood^59^. Inconsistent findings in locomotor regulation were also observed in an axenic *Drosophila* model^13,17^. While our study indicated that GF mice from local vendors exhibited increased locomotion compared to the same strain, sex, and age of SPF mice (Supplementary Fig. 10m), caution is required in interpreting this data. First, the SPF mice utilized in this study were not bred and maintained in gnotobiotic isolators, potentially leading to fundamental differences such as housing, handling, care, and overall mouse husbandry. Second, the GF mice were transferred from gnotobiotic isolators to IVC cages in our institutional animal facility several days before the OFT. This substantial environmental change likely necessitated more time for GF mice to acclimate compared to SPF mice. Third, we overlook the locomotion data, including all SPF mice (Figs. 1g, 3c, 6c; Supplementary Figs. 1e, 1j, 3c, 7k. 10m) alongside the GF mice cohorts (Fig. 6j and Supplementary Fig. 10m). One cohort of GF mice (Fig. 6j) exhibited higher locomotion than SPF mice receiving intraperitoneal saline before the OFT. However, another cohort of GF mice (Supplementary Fig. 10m) demonstrated higher locomotion than most SPF groups. When consolidating the two GF mouse cohorts, only those receiving intraperitoneal saline before the OFT (Fig. 1g and Supplementary Fig. 3c) displayed reduced locomotion. In summary, while GF mice still show a trend toward higher locomotor activity than SPF mice, the difference is not as pronounced as reported in previous studies^14,15,60,61^. Nevertheless, conducting locomotion tests in GF mice alongside well-controlled SPF mice is warranted.

Similarly, depletion of the microbiota by antibiotic(s) did not yield consistent outcomes. Most studies adopting similar ABX recipes did not observe any change in locomotion^20–22^, except for one study that showed increased locomotion^18^ and another study that showed the opposite trend^19^. Another study adopting a single antibiotic in mice showed a reduction in locomotion, which was similar to our findings^62,63^. Both GF and antibiotic methods are powerful ways to eliminate commensal organisms. However, the associations among bacteria, bacterial colonization, and behavior still need to be interpreted with caution. Our results robustly confirmed the locomotor behavior phenotype in gut microbe-depleted adult mice, suggesting that the timepoint for interventions targeting the gut microbiota is crucial for behavioral outcomes in mice.

The bacteria *L. reuteri* has been previously detected in the ileum of neomycin- and metronidazole-treated but not in ampicillin- and vancomycin-treated mice^64^, which is similar to our study (Fig. 5). It is especially intriguing that the mechanisms can involve a single bacterium, *L. reuteri*, exerting an effect on GLP-1 levels. A previous study showed that plasma GLP-1 levels can be altered by the activation of the aryl hydrocarbon receptor, which is the receptor for microbial-mediated tryptophan metabolites^65^. Moreover, the tryptophan derivative indole has been shown to promote enteroendocrine cells to secrete GLP-1^66,67^. *Lactobacillus* strains have been widely demonstrated to metabolize tryptophan into its derivatives, such as indole-3-aldehyde (IAld), indole-3-lactic acid (ILA), and indole-3-acetic acid (IAA)^64,68,69^. Therefore, the metabolism of tryptophan and the production of tryptophan derivatives by colonized *L. reuteri* could be involved in the decrease in GLP-1 levels observed in our study.

Our results demonstrate that the colonization of a single bacterial strain, *L. reuteri*, could not completely suppress GLP-1 levels in mice previously treated with ampicillin (Fig. 6g). This outcome highlights the complexity of the microbial ecosystem in the gut, wherein various microorganisms may possess the capacity to modulate GLP-1 levels. Previous research has identified specific bacterial species, including *Akkermansia muciniphila*, *Staphylococcus epidermidis JA1 strain*, and *Clostridium butyricum*, which have been implicated in promoting the secretion of endogenous GLP-1^70–72^. However, there has been relatively limited exploration of the role of gut microorganisms in inhibiting endogenous GLP-1, with many studies conducted using in vitro models. For instance, experiments involving the co-incubation of Caco2-cells with *Enterococcus faecalis* or *Mitsuokella multiacidus* bacterial supernatants have shown a significant decrease in GLP-1 mRNA abundance^73^. Another study identified isolated species, including *Enterococcus faecalis*, *Clostridium perfringens*, *Clostridium bifermentans*, and *Clostridium butyricum*, which exhibited GLP-1 inhibitory activity in the human cell line NCI-H716^74^. These findings highlight the intricate interplay between gut bacteria and GLP-1 regulation, further emphasizing the need for continued exploration in this area to elucidate the full spectrum of microbial influences on metabolic pathways.

The intricate relationship between GLP-1 and locomotion is compelling, yet establishing a straightforward linear correlation proves elusive. In our investigation, we observed a concurrent hypolocomotion phenomenon in ABX-treated mice (Fig. 1b, c) and ampicillin-treated mice (Fig. 6c) alongside a substantial increase of GLP-1 levels, ranging from 2793.9% to 3735.5% (Figs. 1d and 4c for ABX and ampicillin, respectively). This augmentation in GLP-1 is indeed remarkable, and the magnitude may surpass the threshold necessary to elicit an impact on locomotion. GLP-1, being a multifaceted peptide, exerts a wide array of physiological effects, including its pivotal role in metabolic regulation and satiety^75^. Although elevated GLP-1 levels have been linked to alterations in anxiety, feeding behavior, and glucose homeostasis^44,75,76^, the direct influence of GLP-1 on locomotion remains enigmatic, likely subject to intricate regulatory mechanisms. Conversely, the colonization of *L. reuteri* (Fig. 6f) or the combined colonization of *L. reuteri* and *B. thetaiotaomicron* (Fig. 6j) exhibited the capacity to stimulate locomotor activity in microbiome-depleted mice, accompanied by a reduction in GLP-1 levels ranging from 27.67% to 28.29% (Fig. 6g and k for ampicillin-treated and GF recipients, respectively). Interestingly, colonization by *B. thetaiotaomicron* led to a 25.17% reduction in GLP-1 levels but failed to restore locomotion (Supplementary Fig. 10j-l). These findings prompt speculation regarding the existence of a threshold for the reduction of GLP-1 necessary to promote locomotion, suggesting a finely tuned control mechanism within the gut. This issue, though intriguing, remains complex and warrants in-depth exploration in future investigations.

It is particularly intriguing to note that SPF mice with intact microbiota did not exhibit altered locomotion when vagal signaling was disrupted (Supplementary Fig. 6b). This aligns with previous findings in vagotomized C57BL/6 mice^33,77,78^, Sprague-Dawley rats^79,80^, and Wistar rats^81^, suggesting that the vagus nerve is not required for controlling locomotion when the microbiota is intact. The orchestration of mouse locomotor behavior involves various brain regions such as the motor cortex, basal ganglia, thalamus, mesencephalic locomotor region (MLR), brainstem (pons/medulla), and cerebellum^82,83^. The MLR in the midbrain plays a crucial role in initiating and regulating locomotion speed, comprising three subregions—cuneiform nucleus (CnF), pedunculopontine nucleus (PPN), and mesencephalic reticular formation (mRT)^84,85^. CnF and PPN project glutamatergic signals to the lateral paragigantocellular nucleus (LPGi) in the medulla, regulating high-speed locomotion^84^. On the other hand, the control of locomotion directionality is primarily governed by the glutamatergic projection from the superior colliculus (SC) to the gigantocellular nucleus (Gi)^84^. Circuit-based studies have demonstrated that LPGi can be retrogradely traced to the intestine and nodose ganglion^42^, suggesting an indirect innervation by the vagus nerve. Furthermore, cell-specific transneuronal tracing indicated labeling from the vagal sensory ganglion to dopamine cells in the substantia nigra, but not to brain regions directly regulating locomotion^86^. Vagal signaling can be traced to the parvicellular reticular nucleus (PCRt)^86^, a region receiving GABAergic projections in the central amygdala (CeA), involved in the state-dependent modulation of locomotion, especially feeding- and appetitive-related locomotion^87^. While SDV elevated locomotion in ABX mice (Fig. 3c), it had no impact on locomotion in SPF mice (Supplementary Fig. 6b). Hence, we speculate that the attenuation of vagal signaling in mice by SDV can indirectly influence locomotion, contingent upon the microbiome, nutrient, or appetite states.

Locomotor impairment is one of the symptoms or comorbidities observed in individuals with neurodegenerative diseases^4,5,88^. Clinical and basic studies have revealed dysbiosis of the gut microbiota in subjects with neurodegenerative diseases^89–91^. Recent studies have also demonstrated that the gut microbiota is associated with the progression of neurodegenerative disease pathology^92–95^. Colonizing with the selected bacteria or administering microbial metabolites, such as short-chain fatty acids (SCFAs), could promote or mitigate disease progression^92,95,96^ and motor behavior^92,95,97–99^. Several studies have indicated that agonists of GLP-1R can provide neuroprotective effects^100^ and alleviate neurodegenerative disease^101–103^. GLP-1 is a molecule with multifaceted functions in health and disease^104,105^. Therefore, precise control of the activation or repression of GLP-1 signaling in different body sites will be essential to develop an effective treatment with minimal off-target effects.

Locomotor activity is essential for animal survival. This work highlights the role of the gut microbiome and secreted gut hormones in regulating locomotor behavior. Deficiency in microbes of the gut microbiota impairs locomotion by increasing levels of the gut hormone GLP-1. GLP-1 in the gut signals through the vagal afferent pathway to decrease locomotion. Furthermore, we identified that the specific bacteria *L. reuteri* and *B. thetaiotaomicron* are critical for the suppression of GLP-1 expression and modulation of locomotor behavior. In conclusion, this study determined that gut microbe-mediated GLP-1 signaling regulates locomotor activity via a vagal-dependent pathway (Fig. 7).

## Methods

### Mice

Wildtype C57BL/6JNarl male mice (6-8 weeks) were obtained from the National Laboratory Animal Center (NLAC), Taiwan. C57BL/6JNarl GF mice were bred in the NLAC, Taiwan. The mice were group-housed in ventilated cage (3-5 mice per cage) with a 13 hr light/ 11 hr dark cycle (lights on at 07:00) at 22 ± 1 °C and 55 ± 10% relative humidity in the specific pathogen-free (SPF) animal room in the Laboratory Animal Center, National Cheng Kung University (NCKU). Unless specifically mentioned, all the mice were ad libitum access to chow (Laboratory Autoclavable Rodent Diet PMI 5010, LabDiet) and sterilized drinking water.

Mice were subjected to anesthesia prior to surgical or experimental procedures. Mice were anesthetized using inhaled isoflurane (1-5%, Panion & BF Biotech) in O_2_. Anesthesia induction and maintenance were carefully monitored, and the depth of anesthesia was adjusted to ensure the well-being of the animals throughout the experimental procedures. At the experimental endpoints, mice were euthanized using inhaled isoflurane (5%, Panion & BF Biotech) in O_2_ following approved ethical guidelines. This procedure ensured that animals were under deep anesthesia before euthanasia. Unless otherwise specified, death was confirmed by cervical dislocation to ensure humane and ethical euthanasia.

All the experimental protocols and the animal care were approved by the NCKU Institutional Animal Care and Use Committee (IACUC).

### ABX, sucrose, and selective antibiotic treatment

The ABX treatment was closely following our prior studies^22,106^. Adult mice (8–10 weeks old) were given drinking water for 3 weeks with the cocktail of broad-spectrum antibiotics, including ampicillin (1 g/L, Sigma-Aldrich), vancomycin (0.5 g/L, China Chemical & Pharmaceutical Co., Ltd), neomycin (1 g/L, Fisher Scientific), and metronidazole (0.5 g/L, Acros Organics). Sucrose (10 g/L, Sigma-Aldrich) was added to sweeten the antibiotic water. For the selective antibiotic treatment, single antibiotic was added to the drinking water with the same dosage described above. In the experiment of the SPF mice treated with sucrose water, drinking water with sucrose (10 g/L, Sigma-Aldrich) was given for 3 weeks.

The ABX, sucrose, and selective antibiotic water were then filtered through the 0.22 μm filter (500 ml, Sorfa) for sterilization. Cage and drinking water were changed weekly. Chlorine dioxide (1:5:1, Clidox-S® Base: RO water: Clidox-S® Activator, Pharmacal) was applied to all procedures to avoid bacterial/viral contamination. Normally, the body weight was decreased in the first week of ABX treatment and recovered in the second week of ABX treatment. Behavioral tests and survival surgery were proceeded when the body weight had recovered after ABX treatment^22^. The effect of ABX on the gut microbiome has been shown in our prior work^98^. The 16S rRNA sequencing data have been deposited with links to BioProject accession number PRJNA990939 (ABX) and PRJNA1039541 (selective antibiotic treatment) in the NCBI BioProject database (https://www.ncbi.nlm.nih.gov/bioproject/).

### GLP-1 concentration detection

Blood was collected by cardiac puncture from the isoflurane-anesthetized mice and placed in the serum isolation tube (Sarstedt) with 10 μl DPP4 inhibitor (Millipore). Thirty minutes later, the blood was centrifuged to isolate the serum. The GLP-1 multispecies ELISA kit (Invitrogen) was used for the GLP-1 detection. All the procedures were conducted according to the manufacturer’s protocol. The sera of ABX group were serially diluted to 1:16 due to the upregulation of GLP-1 in ABX mice. For the selective antibiotic treatment, the sera were diluted to 1:8 or 1:16 depending on the type of antibiotic.

### CCK concentration detection

Blood was collected by cardiac puncture from isoflurane-anesthetized mice and placed in the serum isolation tube (Sarstedt). The blood was centrifuged to isolate the serum. The CCK Enzyme Immunoassay kit (RayBiotech) was used for the measurement of CCK. All procedures followed the manufacturer’s protocol. The sera of both the SPF and ABX groups were diluted to 1:16 for detection.

### Drug administration

For GLP-1R agonism, the SPF mice, and the ABX mice received an intraperitoneal injection of 4.2 μg/kg exenatide (Ex-4; Bachem)^107^ dissolved in 0.9% saline 30 minutes before behavior test. For GLP-1R antagonism, the ABX mice, the SPF mice, and the ABX-SDV mice were intraperitoneally injected with the 500 μg/kg exendin (9-39) (Ex(9-39); Bachem)^108^ dissolved in 0.9% saline 30 min before behavior test.

For CCK-induced food intake suppression, the SPF mice were intraperitoneally delivered with 8 μg/kg CCK-8 (Sigma-Aldrich) dissolved in 0.9% saline 30 min before behavior test.

For oxytocin receptor antagonism, 300 µg/kg L-371,257 (Tocris Bioscience) was administrated to mice intranasally. The L-371,257 powder was dissolved in 10% dimethyl sulfoxide (DMSO) in phosphate buffered saline (PBS). The solution was administered two microliters into each nostril 20 min before the behavior test.

### SDV

The SDV procedures were closely following prior studies^22,58,106,109^. Briefly, mice were habituated to the liquid diet (AIN-76A, Research Diets) for two days and were fasted overnight before surgery. All the surgical apparatus, accessories, and surgical site were autoclaved or sterilized before surgery. Mice were anesthetized by isoflurane (1-5%, Panion & BF Biotech) in O_2_ on the heating pad for the entire procedure. Ketoprofen (5 mg/kg, Swiss Pharmaceutical) was given for analgesia and eye ointment was given to avoid eye drying. An abdominal midline and the muscular layer were incised and the liver was gently moved by the cotton swab to expose the stomach and esophagus. The ventral and dorsal vagus nerve trunks were resected by using sharp forceps. In sham operation, the stomach and esophagus were exposed, but the vagal trunks were not resected. The organs were then placed back to their anatomical position. The incised muscular layer was closed by reverse-cutting polyglycolic acid absorbable suture (UNIK) and applied with lidocaine (0.25%, AstraZeneca). The incised skin layer was closed by reverse-cutting chromic catgut absorbable suture (UNIK) and further applied the n-butyl cyanoacrylate adhesive (3 M Vetbond).

After surgery, the mouse was placed into a clean cage with the heating pad underneath. During the recovery, mice were given the liquid diet and gel diet (DietGel 76 A 2 oz, Clear H_2_O) each for two days and then returned to a regular chow diet. The drinking water applied was mixed with Ibuprofen (20 mg/dl, Wyeth-Ayerst) analgesia. Normally, the body weight was decreased in the first week of SDV procedure and recovered in the second week of SDV procedure. Behavioral tests were proceeded when the body weight had recovered from SDV procedure.

The completeness of SDV was validated by fasten-induced food consumption following intraperitoneal CCK-8 injection. The CCK-8-induced anorexia signal is transmitted through the vagus nerve to decrease satiety acutely. The mice were fasted for 20 h and then intraperitoneally injected with 8 μg/kg CCK-8 as previously described. After 2 hours ad libitum feeding, the food intake was recorded and normalized to their body weight^110^.

Mice that underwent vagotomy and displayed a food intake reduction exceeding 30% in comparison to the control group after CCK-8 administration were excluded from the experiment, as this indicated a potential incompleteness of the vagotomy procedure^109,111,112^. Additionally, the exclusion criterion for the percentage of food intake was determined considering their microbiome status. Furthermore, the enlargement of stomach was constantly observed in most vagotomized mice^113^. The retrograde neural tracer FluoroGold (2% w/v, Santa Cruz) was administered intraperitoneally to the mice for neurotracing. The retrograde FluoroGold signal in DMV was quantified for the verification of SDV^47–49^.

### FUS stimulation

To perform ultrasound stimulation, a commercialized 1-MHz FUS probe (A314S-SU-F1.00IN-PTF, focal length = 31.5 mm, OLYMPUS, USA) was driven by a waveform generator (AFG31000, Tektronix, USA) and a radio-frequency power amplifier (150A100D, AR, USA). The FUS stimulation probe and a 5 MHz commercial imaging probe (L7-4, AT5L40B, Verasonics, USA) were integrated by a home-made holder to conduct FUS imaging guided FUS stimulation. A tank filled with degassed water was replaced between FUS probes and the mice head to reduce the acoustic mismatch. There is an opening in bottom of the water tank, which is sealed with a FUS permeable plastic wrap, the mice head is fixed under the plastic wrap, and the ultrasonic coupling gel is used on the head of the mouse.

Mice were first anesthetized with 5% isoflurane (Panion & BF Biotech), after which they were shaved and depilated. 0.1 ml/kg Ketoprofen (2 μl/g, Swiss Pharmaceutical) was injected before surgery to relieve the pain of mice. Mice were mounted on a stereotaxic apparatus with 2% isoflurane (Panion & BF Biotech), and their scalps were thoroughly cleaned with chlorhexidine three times to avoid bacterial infection. The skull of mice was exposed by incising the scalp for FUS imaging guidance FUS stimulation. The 1 MHz FUS stimulation was delivered to DVC (AP = -7.00 mm, ML = ± 0.30 mm, DV = -5.70 mm) with following parameters: 550 kPa peak rarefactional pressure, 10 Hz pulse repetition frequency, 10% duty cycle and duration for 2 min. After finishing the experiment, the mice skull were wiped with lidocaine (0.25%, AstraZeneca), and the mice scalp were glued with Tissue Adhesive (3 M Vetbond). Ibuprofen (20 mg/dl, Wyeth-Ayerst) was added to the drinking water during the recovery from surgery to alleviate pain caused by surgery.

### Brain sample collection and sectioning

Mice were euthanized with isoflurane (5%, Panion & BF Biotech), and subsequently perfused transcardially with PBS and paraformaldehyde (4% w/v, Electron Microscopy Sciences) for brain collection. The brain samples were dissected and post-fixed in 4% w/v paraformaldehyde for 3 days at 4 °C. After 3 days, the brain samples were washed with PBS. The samples were then immersed in PBS with 0.02% sodium azide (Sigma-Aldrich) at 4 °C until sectioning. For the purpose of the c-Fos labeling for neural activation, mice were sacrificed and sampled 80 min after the behavior test. The brain samples were embedded in the 4%UltraPure low melting point agarose (Invitrogen) in PBS on the ice. Then the solidified embedding samples were sectioned coronally for 50 μm by using the vibratome (Leica). The free-floating brain slices were kept in PBS with 0.02% sodium azide (Sigma-Aldrich) at 4 °C until immunohistochemistry staining.

### Immunohistochemistry

The slices were incubated in the blocking buffer (10% horse serum, 0.1% Triton X-100, 0.02% sodium azide in PBS) with the primary antibody at room temperature overnight. The next day, the brain slices were washed by the PBST (0.1% Triton X-100 in PBS) at least 15 min/three times to washout primary antibody. The brain slices were then incubated in the blocking buffer with the secondary antibody that was conjugated with the fluorochrome at room temperature for 2 hours. After the staining, the slices were washed by the PBS for at least 15 min/three times to washout secondary antibody. The slices were either mounted immediately or stored in the PBS with 0.02% sodium azide at 4 °C less than a week. The stained brain slices were mounted on the Superfrost Plus microscope slides (Thermo) with Fluoroshield mounting medium with DAPI (Abcam). Then the microscope cover glasses (Marienfeld) were used to cover the slides. The nail polish was applied on the slide edge for sealing to avoid the leakage. Slides were dried at room temperature overnight for imaging.

Primary antibodies used included goat anti-choline acetyltransferase (ChAT, 1:1000 dilution, AB144P, Millipore) and rabbit anti-c-Fos (1:1000 dilution, 2250 S, Cell Signaling Technology). The secondary antibody conjugated with the fluorochrome included the donkey anti-goat 488 (1:500 dilution) and donkey anti-rabbit 647 (1:500 dilution) (ThermoFisher Scientific).

### Immunohistochemistry staining for colonic GLP-1^+^ cells

Colon tissue was collected from isoflurane-anesthetized mice that were perfused with PBS and paraformaldehyde (4% w/v, Electron Microscopy Sciences). Subsequent to tissue fixation, sections underwent deparaffinization through three sequential immersions in xylene for 10 min each. Following deparaffinization, samples were subjected to rehydration by immersion in two successive changes of absolute alcohol for 5 min each. A stepwise rehydration process in alcohol was then executed, consisting of gradients at 95%, 85%, and 75%, with 2 min allocated to each step. The sections were subsequently rinsed twice in PBS buffer. Hydrogen Peroxide Block (Epredia, TL-125-QHD) was administered to the sections and allowed to incubate for 10 min. For heat-mediated antigen retrieval, tris-EDTA (pH 9.0) treatment was applied for 30 min, followed by a 5-min incubation with immunoblock (Epredia, TL-125-QHD). The anti-GLP-1 antibody (ab22625) was employed at a dilution of 1:25 and subjected to incubation at 37 °C for 1 hour, followed by two sequential rinses in PBS buffer. To facilitate staining development, 30 μl (equivalent to 1 drop) of DAB Chromogen was added to 1.0 ml of DAB Buffer (Epredia, TL-125-QHD), agitated to achieve homogeneity, and subsequently applied to the tissue sections for 10 min. The samples underwent counterstaining with hematoxylin solution for 1 min and were then subjected to a 5-min rinse in running tap water. Following the procedures, the sections were subjected to progressive dehydration, encompassing immersion in 95% alcohol, as well as two successive immersions in absolute alcohol, each lasting 5 min. Lastly, clearance was achieved through immersion in two sequential changes of xylene, with each immersion lasting 5 min. The sections were subsequently mounted employing a xylene-based mounting medium. The results for sectioning, immunostaining, and imaging were obtained from Omics Bio Co., Ltd. (New Taipei City, Taiwan).

Image quantification was performed using the color deconvolution plugin in Fiji software^114^ to separate the DAB channel and the hematoxylin channel. Nuclei were then segmented and transformed into binary masks. Subsequently, the binary reconstruction plugin was applied to calculate the number of DAB-positive nuclei.

### Microscopic imaging and image analysis

The immunostaining was imaged by using the upright THUNDER imaging system (Leica) with LAS X software (Leica). 10X or 20X objective lens was used for specific brainstem area and other brain regions. The positive cell of c-Fos activation was quantified by using the Fiji software (v 2.1.0/1.53c). All c-Fos positive cells quantified were colocalized with DAPI.

### Fecal DNA extraction

The fecal samples were collected by restraining the mice with a single hand and collected with 1.5 ml eppendorf. All the collection was performed in the biosafety cabinet and applied with chlorine dioxide to avoid contamination. All the fecal samples were stored immediately at -80°C until DNA extraction. The Quick-DNA Fecal/Soil Microbe Miniprep kit (Zymo Research) was used for fecal DNA extraction. All procedures were followed by the manufacturer’s protocol. The OD and concentration of extracted DNA were detected by SpectraMax QuickDrop (Molecular Devices) and stored at -80 °C until analysis. The wet weight of the feces was weighed before the extraction for the normalization.

### 16S rRNA sequencing

In 16S rRNA gene sequencing, the sequences were output from 16S rRNA variable region V3-V4 through Illumina Miseq sequencing platform and demultiplexed by Illumina CAVASA 1.8 that obtained from Biotools Co., Ltd. (New Taipei City, Taiwan). The raw data were quality filtered by using QIIME (v1.9.1), and chimeric sequences were removed by UCHIME. We obtained 48,990 ± 3,644 (mean ± s.e.m.) reads of operational taxonomic units (OTUs) in SPF group samples, 39,887 ± 3427 (mean ± s.e.m.) reads in Amp-treated samples, 51,431 ± 4405 (mean ± s.e.m.) reads in Neo-treated samples, 54,438 ± 1620 (mean ± s.e.m.) read in Metro-treated samples, 44,004 ± 2154 (mean ± s.e.m.) reads in Vanco-treated samples. The effective tags were proceeded into OTUs clustering with 97% identity using the UPARSE algorithm in USEARCH software (v7.0.1090). Samples were evened to avoid the deviation caused by different sample sizes. All reads were classified using RDP Classifier (v2.2). The PyNAST database (v1.2), GreenGenes database (gg_13_8), Silva database (v132), NCBI database, and eHOMD database (v15.1) were used in analysis. Alpha diversity and Beta diversity were calculated through QIIME (v1.9.1) including Chao1, ACE, Shannon, Simpson index, and unifrac-based PCoA. Welch’s *t*-test was analyzed by STAMP (v2.1.3). The LDA score was set to 4 in LEfSe analysis. In KEGG functional prediction analysis, PICRUSt (v1.1.1) and GreenGenes database (gg_13_8) were used for the comparison.

### Absolute quantitative PCR for fecal bacteria

The Femto Bacterial DNA Quantification kit (Zymo Research) was used for the absolute bacterial quantitative PCR. The DNA samples were 1:100 diluted. All procedures followed the manufacturer’s protocol.

### Relative quantitative PCR

The Power SYBR Green PCR Master Mix (ThermoFisher Scientific) kit was used for the relative qPCR. The DNA samples were diluted to 10 ng/μl, depending on the original concentration. The total reaction volume was 25 μl, containing: 12.5 μl Power SYBR Green PCR Master Mix, 0.5 μl of each bacterial primer (including the forward and reverse), 6.5 μl of nuclease-free water, and 5 μl of DNA. The qPCR was performed in duplicates by using a 96-well plate (ThermoFisher Scientific).

16S primer (F: 5’-ACTCCTACGGGAGGCAGCAGT-3’; R: 5’-ATTACCGCGGCTGCTGGC-3’) was used for the normalization of the expression levels between different targets. Primers used to detect the species of bacteria, including the *Lactobacillus (L.) reuteri* (F: 5’-GAAGATCAGTCGCAYTGGCCCAA-3’; R: 5’-TCCATTGTGGCCGATCAG-3’) and *Bacteroides (B.) thetaiotaomicron* (F: 5’-TACAATTGCCACAGTACGGAACA-3’; R: 5’-GCTGACGAACGATGACCATAGTTA-3’).

### Bacterial colonization

*L. reuteri* (DSM17938) stocks were streaked on the MRS agar plate (BD Difco) and cultured aerobically at 37 °C. Inoculate the single colony in 5 ml MRS broth (BD Difco) aerobically at 37 °C. The overnight culture in 5 ml broth was then inoculated to 20 ml MRS broth on the next day to yield the consistent colony-forming unit (CFU). Afterward, the overnight culture in 20 ml broth was centrifuged and washed the cultured pellet in pre-reduced PBS with 1.5% sodium bicarbonate (GeneDireX). Finally, the bacterial pellet was thoroughly dispersed with PBS and ready to use for bacterial treatment.

*B. thetaiotaomicron* (DSM 2255) was streaked on the blood agar plates and cultured anaerobically at 37 °C. The single colony was washed and resuspended in PBS for bacterial colonization in GF mice. A single colony was inoculated into 5 ml of Tryptic Soy Broth (BD Difco) under anerobic conditions at 37 °C for an overnight culture lasting 3 days. Afterward, the broth was centrifuged and the cultured pellet was washed by pre-reduced PBS. The bacterial pellet was then resuspended in PBS for bacterial colonization in the ampicillin-treated mice.

The SPF mice were treated with ampicillin (1 g/L, Sigma-Aldrich) for three weeks and received the *L. reuteri* bacterial liquid. Briefly, the experimental group received live bacteria (~1 × 10^9^ CFU/ml) in sterilized drinking water, while the control group received PBS as vehicle. The water was changed every two days. For the mice received *B. thetaiotaomicron*, each mouse received 200 µl of bacteria (~1 × 10^9^ CFU/ml) for 6 times in three weeks.

Eight-week old C57BL/6JNarl GF male mice were colonized with single oral gavage (0.2 ml/mice) of the *L. reuteri* and *B. thetaiotaomicron* bacterial mixture liquid. The behavioral test was proceeded 4 weeks after gavage.

### Behavior

#### General guideline for behavioral test

The behavior experiments were conducted within the 8AM-13PM. Mice were habituated to the testing room at least 40 min before behavior test. All behavior tests were performed at 8-14 weeks of age. For the behavioral experiment of SPF mice injected Ex-4 and ABX mice injected Ex(9- 39), mice were tested for OFT at 10 weeks of age, EPM at 11 weeks of age, and novel object recognition test at 12 weeks of age. Finally, the mice were tested again for the OFT without the injection of Ex-4 or Ex(9-39) a week after novel object recognition test and the brains were collected 80 min after OFT for c-Fos staining. For the behavioral experiments of SPF-Sham, ABX-Sham and ABX-SDV, mice were tested for OFT at 11 weeks of age, EPM at 12 weeks of age, and motor function test (wire hanging test, ledge test, and hindlimb clasping test) at 13 weeks of age. Finally, the mice were tested the CCK-injected food intake for SDV validation. For the behavioral experiment of SPF-Sham and SPF-FUS, mice were tested OFT at 9 weeks of age, CCK-8 injected food intake at 10 weeks of age, saline-injected 11 weeks of age, and motor function test (wire hanging test, ledge test, and hindlimb clasping test, and rotarod test) at 12 weeks of age. The mice were injected CCK again a week after motor function test and the samples were collected after 80 min. The light intensity for the testing environment of OFT (center 10 lux, peripheral 6 lux), EPM (open arm 35 lux, close arm 20 lux), Novel cage locomotion test (15 lux), wire hanging test (600 lux), ledge test (600 lux), hindlimb clasping test (600 lux), novel object recognition test (15 lux). The behavior testing chamber was cleaned with chlorine dioxide for sterilization, then wiped with 70% ethanol to eliminate the odor from the prior tested mouse. After cleaning, the chamber was air-dried for 5 minutes before placing the next animal.

#### OFT

The OFT is a commonly used experiment to measure the general locomotor activity, novel environment exploration, and anxiety-like behavior^11^. Mice were placed in an acrylic-made open square arena (48 × 48 cm) and allowed to explore for 10 minutes freely. The video was recorded by the ceiling mounted camera and analyzed by Ethovision XT 14/15 (Noldus Information Technology), including the distance moved and duration of stay. The center of the arena was defined as the center zone (24 × 24 cm) and the peripheral zone was defined as 6 cm inward from the chamber’s boundary.

#### Novel cage locomotion test

Mice were placed in a clean and sterilized cage and allowed them to freely explore 10 min. The video was recorded by the ceiling mounted camera and analyzed the distance moved by Ethovision XT 14/15 (Noldus Information Technology).

#### EPM test

The EPM is a test to assess anxiety-like behavior and innate fear^115^. This maze is composed of a “+“ shape with two opposite directions of opened, closed arms and a center area (5 × 5 cm). Both opened arms and closed arms are 30 cm in length and 5 cm in width, but only the closed arms had the 15 cm heighten walls around. The whole maze was set up 55 cm above the floor to induce the propensity of mice acrophobia. Mice were allowed to freely explore the maze for 5 min. The video was recorded and analyzed the distance moved, frequency of entry, and duration in opened and closed arms by Ethovision XT 14/15 (Noldus).

#### Wire hanging test

The wire hanging test was adapted from previously described protocols^116^.A wire cage top was placed upon three stacked cages on each side (height: 40 cm). Mouse was placed on the wire cage top first. Then the wire cage top was inverted to allow the mice to grab the wire and suspend. The latency of mouse fell onto the bedding was recorded. The score was defined based on the latency time. Score 1: 1–10 s latency; Score 2: 11–25 s latency; Score 3: 26–60 s latency; Score 4: 61–90 s latency; Score 5: > 90 s latency.

#### Ledge test

The ledge test is a specific test to evaluate the ability of motor coordination in mice^117^. Mouse was placed on the ledge of the cage. The experimenter observed detailed motor features when the mouse moved forward and backward along the ledge. The score was assigned from 0-3 based on their performance. Score 0: The standard and adequate motor coordination performance. The mouse was observed walking along the ledge and back into the cage without losing balance. Score 1: The mouse loses its balance while walking on the ledge but is coordinated. Score 2: The mouse could not effectively use its hindlimbs or back into the cage by landing on head rather than paws. Score 3: Mouse falls off the ledge, shakes, and refuses to move on the ledge.

#### Hindlimb clasping test

Mouse tail was grabbed and suspended for 10–15 s. The experimenter observed in the orientation of abdominal area where the mouse hindlimb exposed. If the hindlimbs stretched outward constantly without any abnormal movement, a score of 0 was assigned. If the hindlimb were withdrawing toward the abdomen, 1-3 was assigned depending on the level of severity. Score 1: One hindlimb is stretched toward the abdomen for more than half of the time suspension. Score 2: Two hindlimbs are stretched toward the abdomen for more than half of the time suspension. Score 3: Two hindlimbs are entirely stretched toward and touching the abdomen for more than half of the time suspension^117^.

#### Novel object recognition test

Novel object recognition test was adapted from previously described protocols^118^. The novel object recognition test is used to measure the learning and memory of mice. This test includes three phases: the habituation phase, the training phase, and the testing phase. In the habituation stage, mouse was placed in an acrylic-made open square arena (48 × 48 cm) and allowed to freely explore for 5 min. To minimize the confounding effect produced by circadian rhythm, it is crucial to execute this procedure for the same mice at the same time point every day for three days. Two different objects were applied in the training phase: the LEGO bricks (8 × 3.5 cm) and Falcon tissue culture flask filled with sand (9.5 × 5.5 × 2.5 cm). The pre-test indicated that mice showed the similar preference to these two objects. In the training phase, the experimenter placed two identical objects in the north-west corner and the south-east corner of the square arena to allow the mouse to explore for 10 min. Then the mouse was returned to their home cage. Before the testing phase, the exploration time of the two identical objects (familiar object) was measured. Four hours later, one familiar object that the mice explored less previously was switched to a novel object to avoid the spatial preference. Mouse was then re-introduced into the testing arena for 10 min. For data collection, the mice were excluded if the total exploration time of the two objects in the testing phase did not exceed 20 s. The formula of the discriminate index: (Time to explore the novel object—Time to explore the familiar object)/ (Time to explore the novel object + time to explore the familiar object).

#### Rotarod

The rotarod test is used to evaluate the motor function, coordination, and balance of animals^119^. During the training phase, there are 4 trials each day with 5-min intervals between each trial, conducted over 3 consecutive days. The mice were placed on a rotating rod (Ugo Basile) that maintained a constant speed of 24 rpm, and each trial lasted for 60 s for motor learning test. The latency of mouse fall from the rod was recorded.

In the testing phase, the mice were placed on the rod, and the speed was linearly increased from 5 to 40 rpm during the first minute. It then remained stable at 40 rpm for an additional 2 min. The latency of mouse fall from the rod was recorded.

### Statistical analysis

The statistical data were presented as mean ± SEM. The number of n represents the number of biological replicates. In Fig. 3c, two ABX-SDV mice were excluded due to weakness or death after surgery. In Fig. 3f, three ABX-SDV mice were excluded due to food intake reduction exceeding 30% in comparison to the control group after CCK-8 administration. A two-tailed unpaired *t*-test was used to compare the means of two independent groups. Data with more than two independent groups were analyzed by one-way ANOVA with Bonferroni’s multiple comparison post-hoc test. Data with two factors and one of the factors was repeated were analyzed by two-way repeated measure ANOVA with Bonferroni’s multiple comparison post-hoc test. There were three ABX-SDV mice excluded due to weakness or death after SDV. All data were analyzed by Prism 9 (GraphPad). The significance between groups was decided by the *P*-value. The statistically significance between groups was determined when the *P*-value is smaller than 0.05. The groups were considered as different while having the asterisk. The asterisks in the comparison: *P < 0.05, **P < 0.01, ***P < 0.001, ****P < 0.0001.

### Reporting summary

Further information on research design is available in the Nature Research Reporting Summary linked to this article.

### Supplementary information

Supplementary Figures

Reporting Summary

## Data Availability

The data that support the findings of this study are available from the corresponding author upon request. The data for 16S rRNA gene sequencing have been deposited with links to BioProject accession number PRJNA1039541 in the NCBI BioProject database (https://www.ncbi.nlm.nih.gov/bioproject/) and will be accessed upon published.
