# Supplementary material for: The gut microbiota modulate locomotion via vagus-dependent glucagon-like peptide-1 signaling
Source: NPJ Biofilms Microbiomes. 2024 Jan 16;10:2. doi: 10.1038/s41522-024-00477-w (PMC10791613; doi:10.1038/s41522-024-00477-w)
Supplement: Supplementary file 1 — Supplementary Figures [file 41522_2024_477_MOESM1_ESM.pdf]

Supplementary Figures and Figure Legends

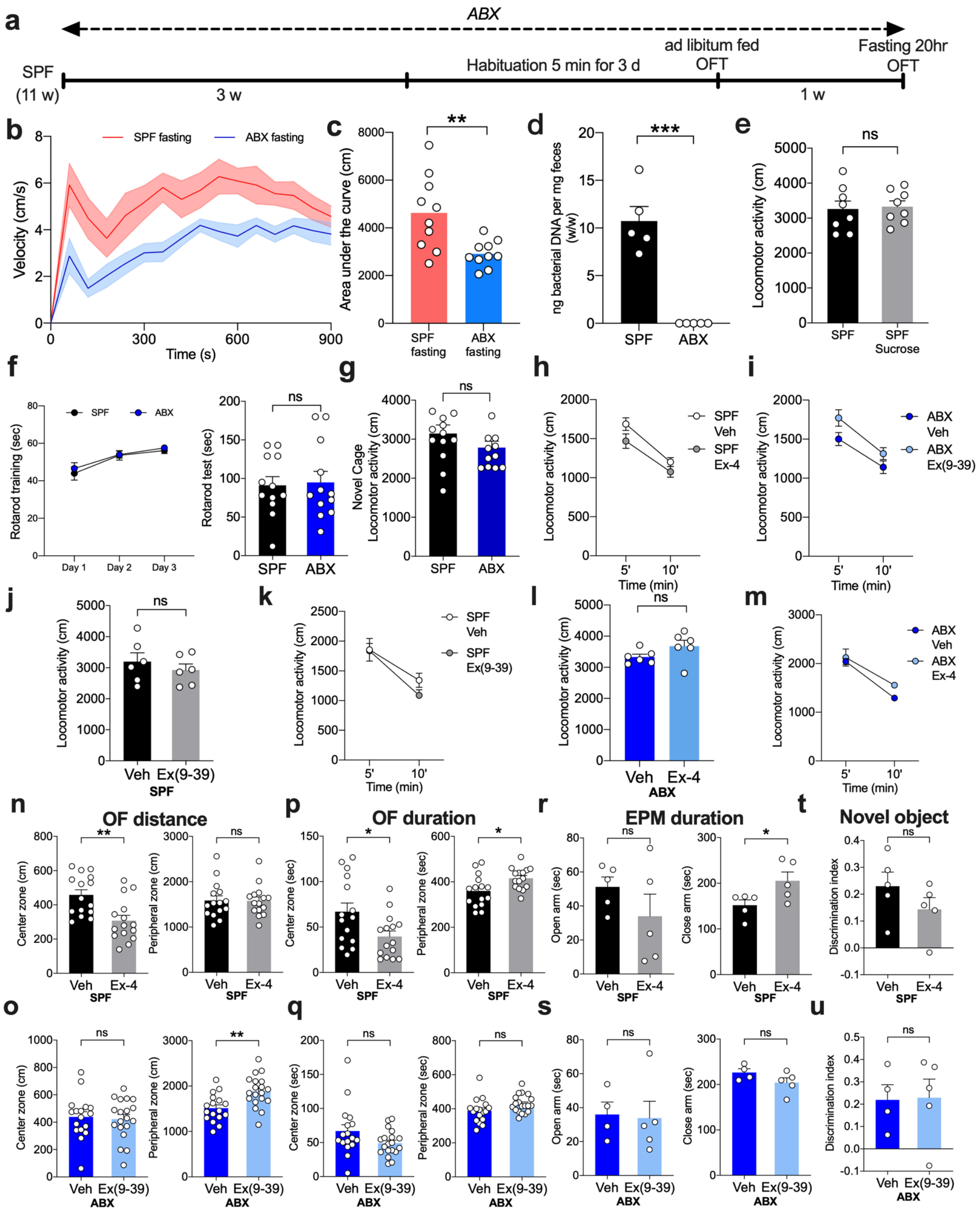

Supplementary Figure 1. The alteration of locomotor activity by ABX was not due to the anxiety-like behavior or cognitive impairment.

(a) Timeline schematic of the habituation, fasting, and OFT. The mice after three weeks ABX treatment were habituated in the OFT paradigm 5 minutes for three days. Then tested the OFT in ad libitum fed and 20 hours fasting condition.

(b) Velocity during the OFT in SPF-fasting mice and ABX-fasting mice (n = 10).

(c) Area under the curve of the velocity-time graph represents the distance moved in SPF-fasting mice and ABX-fasting mice (n = 10).

(d) Absolute fecal bacteria DNA was quantified in SPF and ABX groups. The bacteria DNA was significantly reduced in feces of ABX mice (n = 5).
(e) Locomotor activity was tested using the OFT paradigm in SPF-Veh and SPF-Sucrose mice (n = 8).
(f) Rotarod test in training phase (left) and testing phase (right) (n = 12).
(g) Distance moved was tested using the novel cage in SPF and ABX mice. Locomotor activity was analyzed in whole arena (n = 12-13).
(h) Locomotor activity was tested using the OFT paradigm in 5 minutes bin in SPF-Veh and SPF-Ex-4 mice (n = 15).
(i) Locomotor activity was tested using the OFT paradigm in 5 minutes bin in ABX-Veh and ABX-Ex(9-39) mice (n = 17-19).
(j) Locomotor activity was tested using the OFT paradigm in SPF-Veh and SPF-Ex(9-39) mice (n = 6).
(k) Locomotor activity was tested using the OFT paradigm in 5 minutes bin in SPF-Veh and SPF-Ex(9-39) mice (n = 6).
(l) Locomotor activity was tested using the OFT paradigm in ABX-Veh and ABX-Ex-4 mice (n = 6).
(m) Locomotor activity was tested using the OFT paradigm in 5 minutes bin in ABX-Veh and ABX-Ex-4 mice (n = 6).
(n) Locomotor activity in center zone (left) and peripheral zone (right) of OFT was in SPF-Veh and SPF-Ex-4 mice (n = 15).
(o) Locomotor activity in center zone (left) and peripheral zone (right) of OFT was in ABX-Veh and ABX-Ex(9-39) mice (n = 17-19).
(p) Time spent in center zone (left) and peripheral zone (right) of OFT was in SPF-Veh and SPF-Ex-4 mice (n = 15).
(q) Time spent in center zone (left) and peripheral zone (right) of OFT was in ABX-Veh and ABX-Ex(9-39) mice (n = 17-19).
(r) Time spent in open arm (left) and close arm (right) of EPM was in SPF-Veh and SPF-Ex-4 mice (n = 5).
(s) Time spent in open arm (left) and close arm (right) of EPM was in ABX-Veh and ABX-Ex(9-39) mice (n = 4-5).
(t) Discrimination index of NORT was in SPF-Veh and SPF-Ex-4 mice (n=5).
(u) Discrimination index of NORT was in ABX-Veh and ABX-Ex(9-39) mice (n=4).

Data represent mean ± SEM. Data analyzed by two-tailed unpaired *t*-test (c, d, e, f-right, g, j, l, n-u), two-way ANOVA with repeated measures with Bonferroni's multiple comparison post-hoc test (f-left, h, i, k, m). * $P < 0.05$; ** $P < 0.01$; ns: no significant.

SPF: specific-pathogen-free; ABX: antibiotic cocktail; Veh: vehicle; Ex-4: exenatide; Ex(9-39): exendin(9-39); OFT: open-field test; EPM: elevated plus maze; NORT: novel object recognition test; w: week; d: day; hr: hour; min: minute.

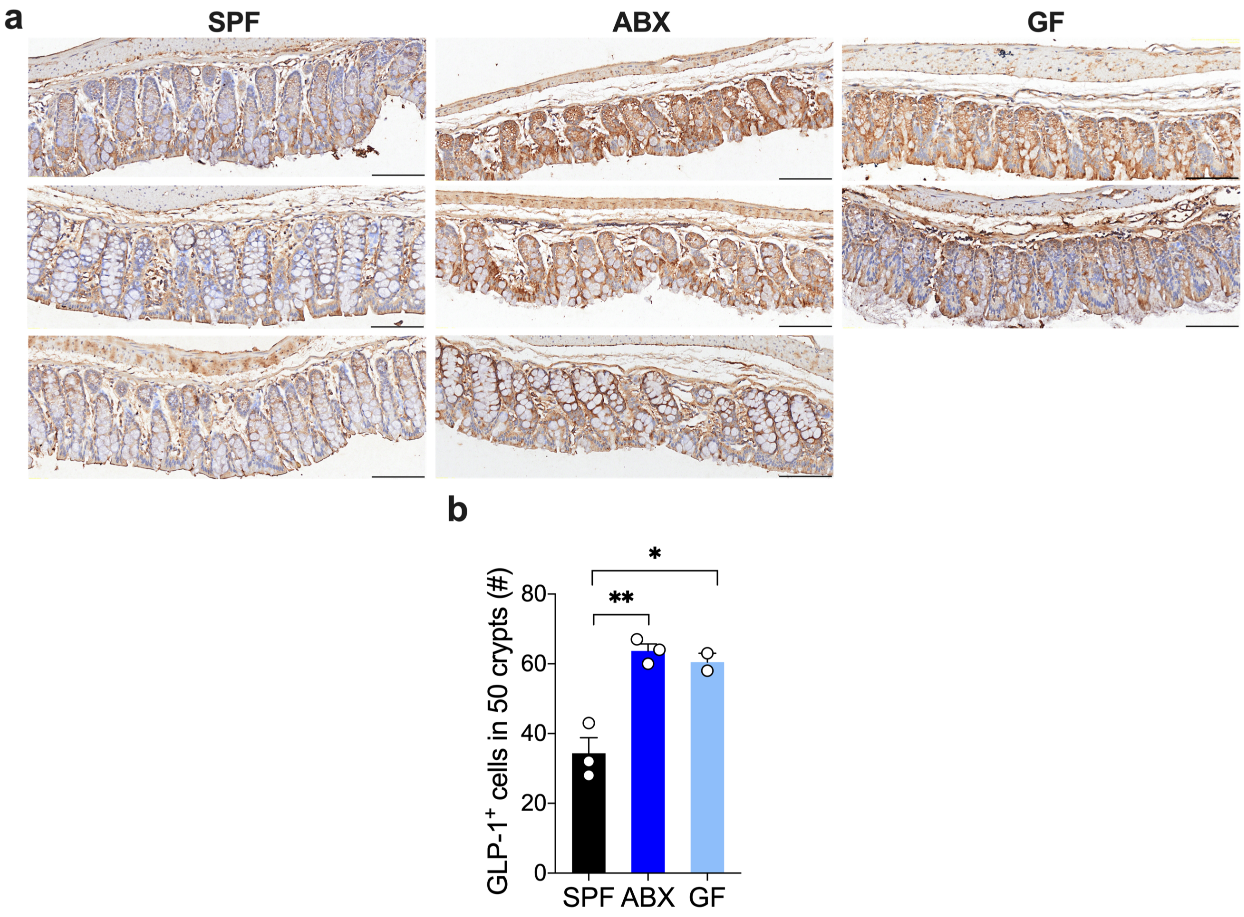

Supplementary Figure 2. Distribution of GLP-1⁺ cells in the colon in ABX and GF mice.
(a) Images of GLP-1 staining in colonic sections from SPF, ABX, and GF mice. Scale bar = 100 µm.
(b) Quantification of GLP-1+ cells in the colon of SPF, ABX, and GF mice (n = 2-3).

Data represent mean ± SEM. Data analyzed by unpaired *t*-test (b). * $P < 0.05$; ** $P < 0.01$.

SPF: specific-pathogen-free; ABX: antibiotic cocktail; GF: germ-free.

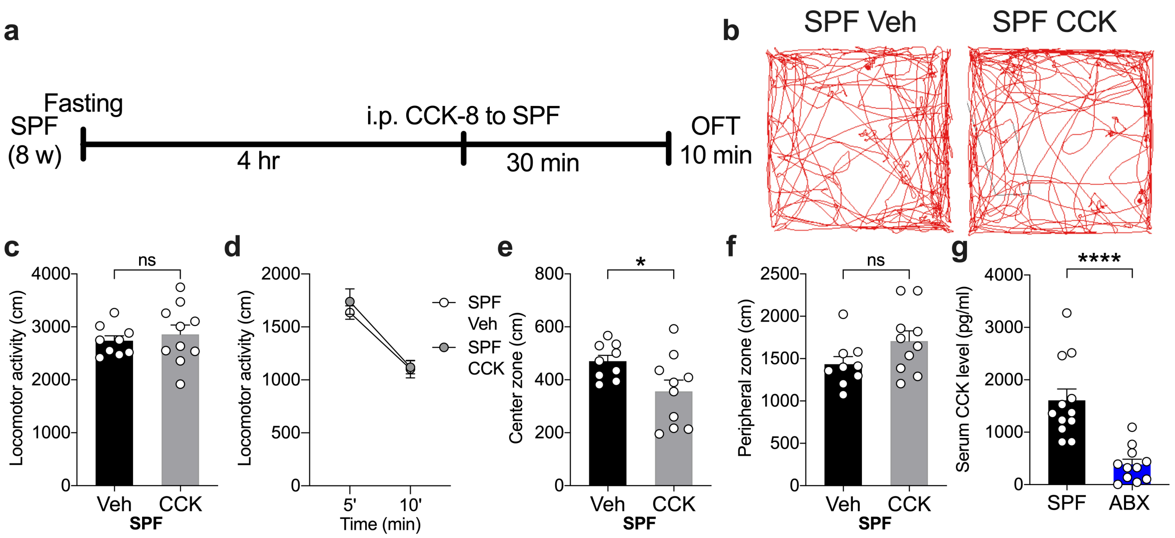

Supplementary Figure 3. No effect was produced by cholecystokinin-8 (CCK-8) on the locomotion in SPF mice.

(a) Timeline schematic of the fasting, CCK-8 injection, and OFT. The mice were fasted 4 hours prior the injection of the CCK-8 and tested for OFT 30 minutes after injection.
(b) Representative images of trajectories of OFT in SPF-Veh and SPF-CCK mice.
(c) Locomotor activity was tested using the OFT paradigm (n = 9-10).
(d) Locomotor activity was tested using the OFT paradigm in 5 minutes bin in SPF-Veh and SPF-CCK mice (n = 9-10).
(e) Locomotor activity in center zone of OFT was in SPF-Veh and SPF-CCK mice (n = 9-10).
(f) Locomotor activity in peripheral zone of OFT was in SPF-Veh and SPF-CCK mice (n = 9-10).
(g) Measurement of serum CCK concentrations in SPF, ABX mice (n = 11-12).

Data represent mean ± SEM. Data analyzed by two-tailed unpaired t-test (c, e, f, g); two-way ANOVA repeated measures with Bonferroni's multiple comparison post-hoc test (d). * $p \leq 0.05$; ns: no significant.

SPF: specific-pathogen-free; CCK-8: cholecystokinin-8; CCK: cholecystokinin; OFT: open-field test; Veh: vehicle; w: week; hr: hour; min: minute.

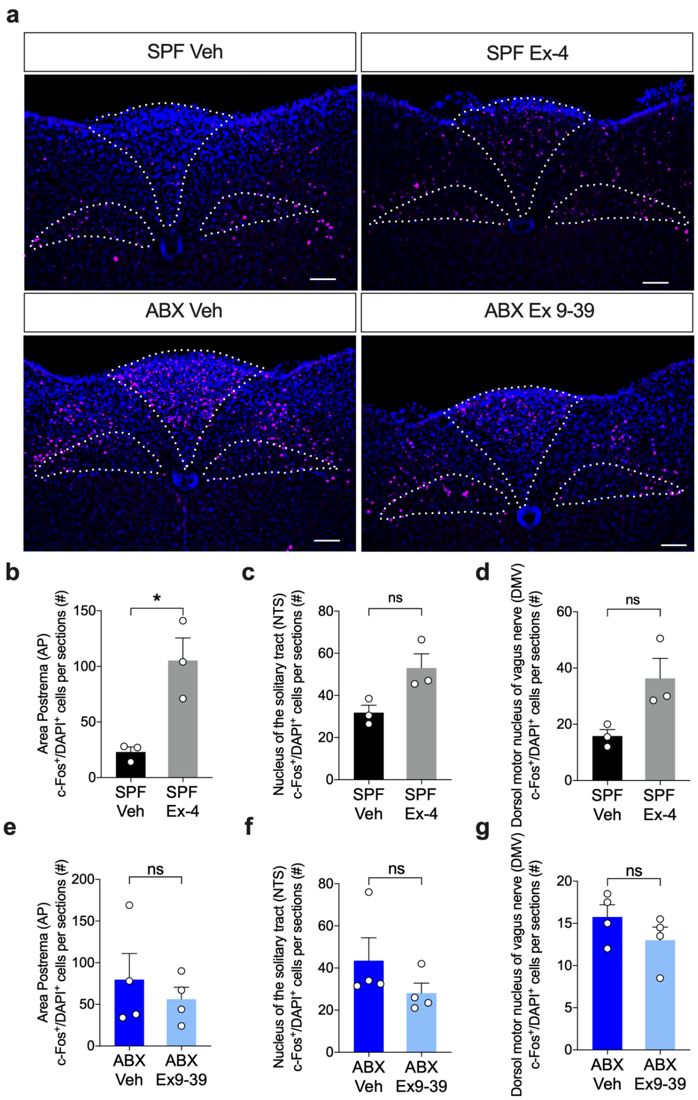

Supplementary Figure 4. Expression of c-Fos+ cells were detected in vagal ascending brain regions in SPF-Ex-4 mice after OFT.
(a) Representative images of c-Fos staining in brain sections from SPF-Veh, SPF-Ex-4, ABX-Veh, and ABX-Ex(9-39) mice after OFT. Scale bar = 100 μm.
(b-d) Quantification of c-Fos+ cells in the vagal ascending brain regions of SPF-Veh and SPF-Ex-4 mice after OFT (n = 3).
(e-g) Quantification of c-Fos+ cells in the vagal ascending brain regions of ABX-Veh and ABX-Ex(9-39) mice after OFT (n = 4).

Data represent the mean ± SEM. Data were analyzed by two-tailed unpaired *t test* (b, c, d, e, f, g). * $P < 0.05$; ns: not significant.

SPF: specific-pathogen-free; ABX: antibiotic cocktail; Veh: vehicle; Ex-4: exenatide; Ex(9-39): exendin(9-39); AP: area postrema; NTS: nucleus of the solitary tract; DMV: dorsal motor nucleus of vagus nerve.

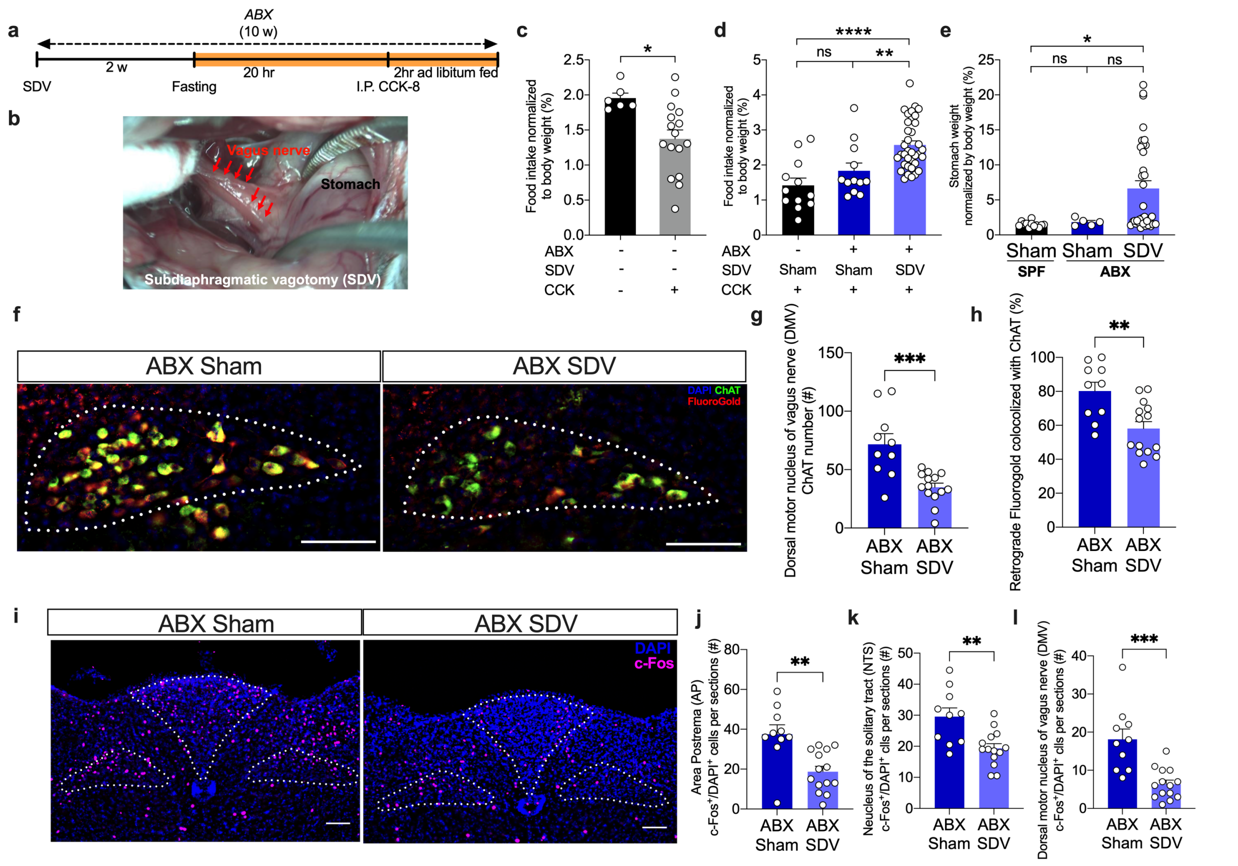

Supplementary Figure 5. Verification of SDV procedure in ABX mice.

(a) SDV procedure, CCK-8-induced anorexia, and food intake measurement. SDV procedure was executed two weeks after broad-spectrum ABX treatment. The mice were given additional two weeks to recover from SDV.

(b) Representative image of SDV procedure. Arrows indicate the subdiaphragmatic vagus nerve aligned with esophagus.

(c) Food intake normalized to body weight was reduced in surgery naïve CCK-injected control (SPF) mice (n = 6-16).

(d) The completeness of SDV was validated by fasten-induced food consumption for 2 hours following by intraperitoneal CCK-8 injection. Food intake normalized to body weight was increased in CCK-injected ABX-SDV mice (n = 12-36).

(e) Stomach weight normalized to body weight was measured in SPF-Sham, ABX-Sham, ABX-SDV mice (n = 5-34).

(f) Representative images of Fluorogold and ChAT staining in brain sections from ABX sham and ABX SDV mice. Scale bar = 100 µm.

(g) Quantification of ChAT+ cells in the DMV of ABX sham and ABX SDV (n = 10-14).

(h) Quantification of ChAT and Fluorogold double positive cells in the DMV of ABX sham and ABX SDV (n = 10-14).

(i) Representative images of c-Fos staining in brain sections from ABX sham and ABX SDV mice. Scale bar = 100 µm.

(j-l) Quantification of c-Fos+ cells in the vagal ascending brain regions of ABX sham and ABX SDV mice (n = 10-14).

Data represent mean ± SEM. Data analyzed by two-tailed unpaired *t*-test (c, g, h, j, k, l) and one-way ANOVA (d, e) with Bonferroni's multiple comparison post-hoc test. * $P < 0.05$; ** $P < 0.01$; *** $P < 0.001$; **** $P < 0.0001$; ns: no significant.

SPF: specific-pathogen-free; ABX: antibiotic cocktail; SDV: subdiaphragmatic vagotomy; CCK-8: cholecystokinin-8; CCK: cholecystokinin; ChAT: choline acetyltransferase; DMV: Dorsal motor nucleus of vagus nerve I.P.: intraperitoneal; w: week; hr: hour.

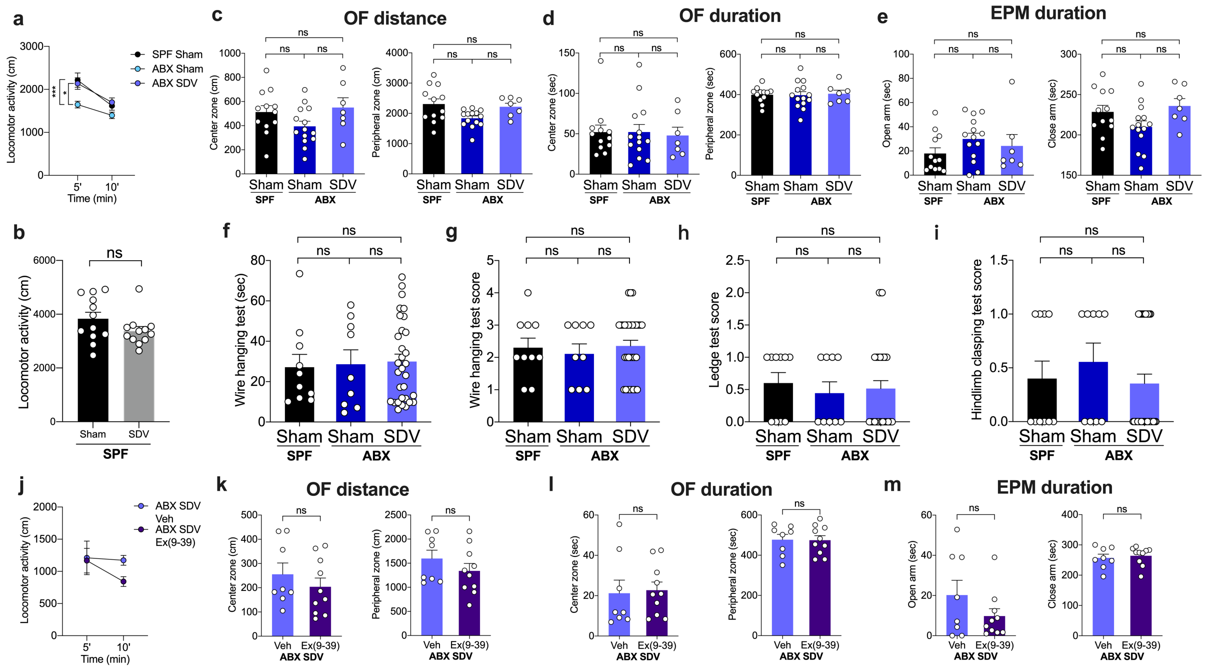

Supplementary Figure 6. SDV procedure did not produce effect on anxiety-like behavior and motor function in ABX mice.

(a) Locomotor activity was tested using the OFT paradigm in 5 minutes bin in SPF-Sham, ABX-Sham, ABX-SDV mice (n = 7-14).

(b) Locomotor activity was tested using the OFT paradigm in SPF-Sham (n = 12, data from Fig. 3c) and SPF-SDV mice (n = 12).

(c) Locomotor activity in center zone (left) and peripheral zone (right) of OFT was in SPF-Sham, ABX-Sham, ABX-SDV mice (n = 7-14).

(d) Time spent in center zone (left) and peripheral zone (right) of OFT was in SPF-Sham, ABX-Sham, ABX-SDV mice (n = 7-14).

(e) Time spent in open arm (left) and close arm (right) of EPM was in SPF-Sham, ABX-Sham, ABX-SDV mice (n = 7-14).

(f) Hanging time (latency to fall) was tested using the wire hanging test paradigm in SPF-Sham, ABX-Sham, ABX-SDV mice (n = 9-31).

(g) Hanging time (score) was tested using the wire hanging test paradigm in SPF-Sham, ABX-Sham, ABX-SDV mice (n = 9-31).

(h) Score of the ledge test in SPF-Sham, ABX-Sham, ABX-SDV mice (n = 9-31).

(i) Score of the hindlimb clasping test in SPF-Sham, ABX-Sham, ABX-SDV mice (n = 9-31).

(j) Locomotor activity was tested using the OFT paradigm in 5 minutes bin in ABX-SDV-Veh and ABX-SDV-Ex(9-39) mice (n = 8-10).

(k) Locomotor activity in center zone (left) and peripheral zone (right) of OFT was in ABX-SDV-Veh and ABX-SDV-Ex(9-39) mice (n = 8-10).

(l) Time spent in center zone (left) and peripheral zone (right) of OFT was in ABX-SDV-Veh and ABX-SDV-Ex(9-39) mice (n = 8-10).

(m) Time spent in open arm (left) and close arm (right) of EPM was in ABX-SDV-Veh and ABX-SDV-Ex(9-39) mice (n = 8-10).

Data represent mean ± SEM. Data analyzed by two-tailed unpaired *t*-test (k, l, m), one-way ANOVA (c, d, e, f, g, h, i), and two way ANOVA with repeated measures (a, j) with Bonferroni's multiple comparison post-hoc test. * $P < 0.05$; ** $P < 0.01$; *** $P < 0.001$; **** $P < 0.0001$; ns: no significant.

SPF: specific-pathogen-free; ABX: antibiotic cocktail; SDV: subdiaphragmatic vagotomy; Ex(9-39): exendin(9-39).

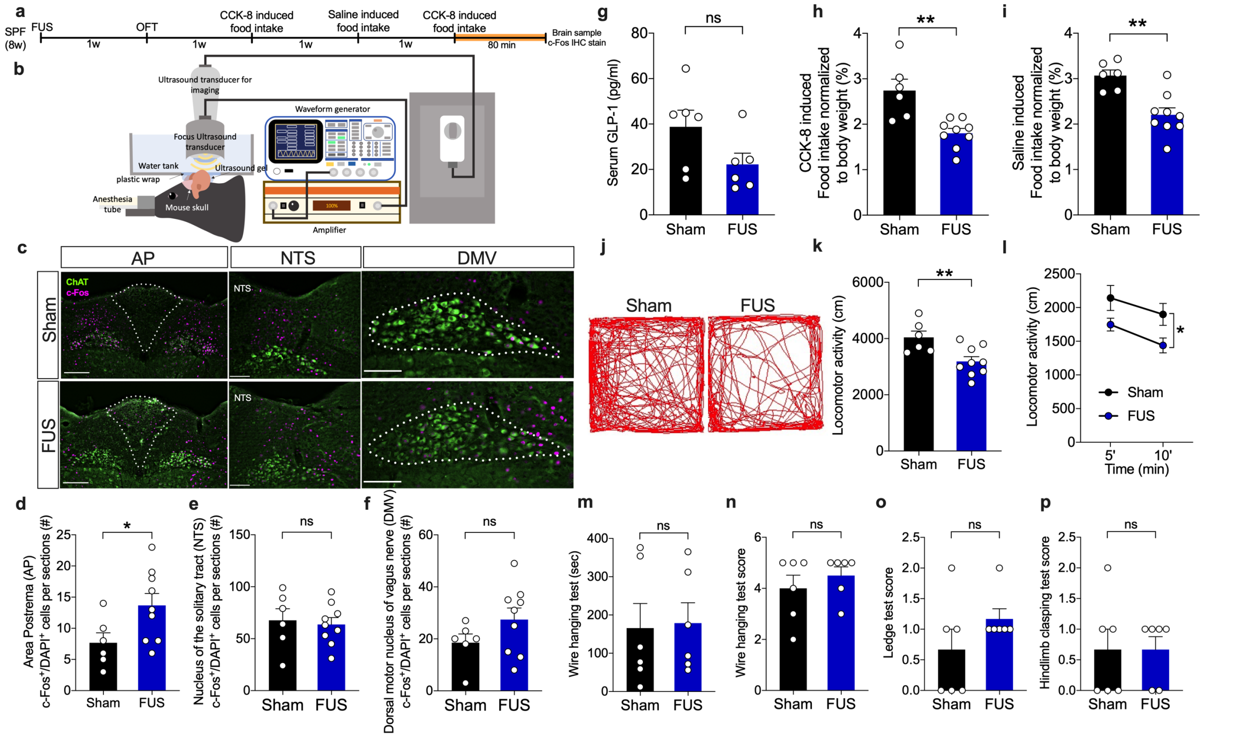

Supplementary Figure 7. Focused ultrasound stimulation (FUS) on the brainstem of SPF mice induces the hypolocomotion phenotype.

(a) Timeline schematic of the FUS, OFT, CCK-8- and saline-induced food intake. The mice were tested for OFT one week after FUS.

(b) Diagram of brainstem FUS in mice.

(c) Representative images of c-Fos staining in brain sections from SPF-Sham and SPF-FUS mice after CCK-8 induced anorexia food intake. Scale bar = 100 μm

(d) Quantification of c-Fos+ in SPF-Sham and SPF-FUS mice after OFT in AP (n =6-9).

(e) Quantification of c-Fos+ cells in NTS of SPF-Sham and SPF-FUS mice after OFT (n =6-9).

(f) Quantification of c-Fos+ and ChAT+ cells in DMV of SPF-Sham and SPF-FUS mice after OFT (n =6-9).

(g) Measurement of serum GLP-1 concentrations showed no difference between SPF-Sham and SPF-FUS mice (n = 6).

(h) Food intake normalized to the body weight was reduced in SPF-FUS mice after CCK-8 injection (n = 6-9).

(i) Food intake normalized to the body weight was reduced in SPF-FUS mice after saline injection (n = 6-9).

(j) Representative images of trajectories of OFT in SPF-Sham and SPF-FUS mice.

(k) Locomotor activity was tested using the OFT paradigm in SPF-Sham and SPF-FUS mice (n = 6-9).

(l) Locomotor activity was tested using the OFT paradigm in 5 minutes bin in SPF-Sham and SPF-FUS mice (n = 6-9).

(m) Hanging time (latency to fall) was tested using the wire hanging test paradigm in SPF-Sham and SPF-FUS mice (n = 6).

(n) Hanging time (score) was tested using the wire hanging test paradigm in SPF-Sham and SPF-FUS mice (n = 6).

(o) Score of the ledge test in SPF-Sham and SPF-FUS mice (n = 6).
(p) Score of the hindlimb clasping test in SPF-Sham and SPF-FUS mice (n = 6).

Data represent mean ± SEM. Data analyzed by two-tailed unpaired *t*-test (d-i, k, m-p), two-way ANOVA with repeated measures with Bonferroni's multiple comparison post-hoc test (l). * $P < 0.05$; ** $P < 0.01$; ns: no significant.

SPF: specific-pathogen-free; FUS: focused ultrasound; CCK-8: cholecystokinin-8; OFT: open-field test; w: week; min: minute; AP: area postrema; NTS: nucleus of the solitary tract; DMV: dorsal motor nucleus of vagus nerve; ChAT: choline acetyltransferase.

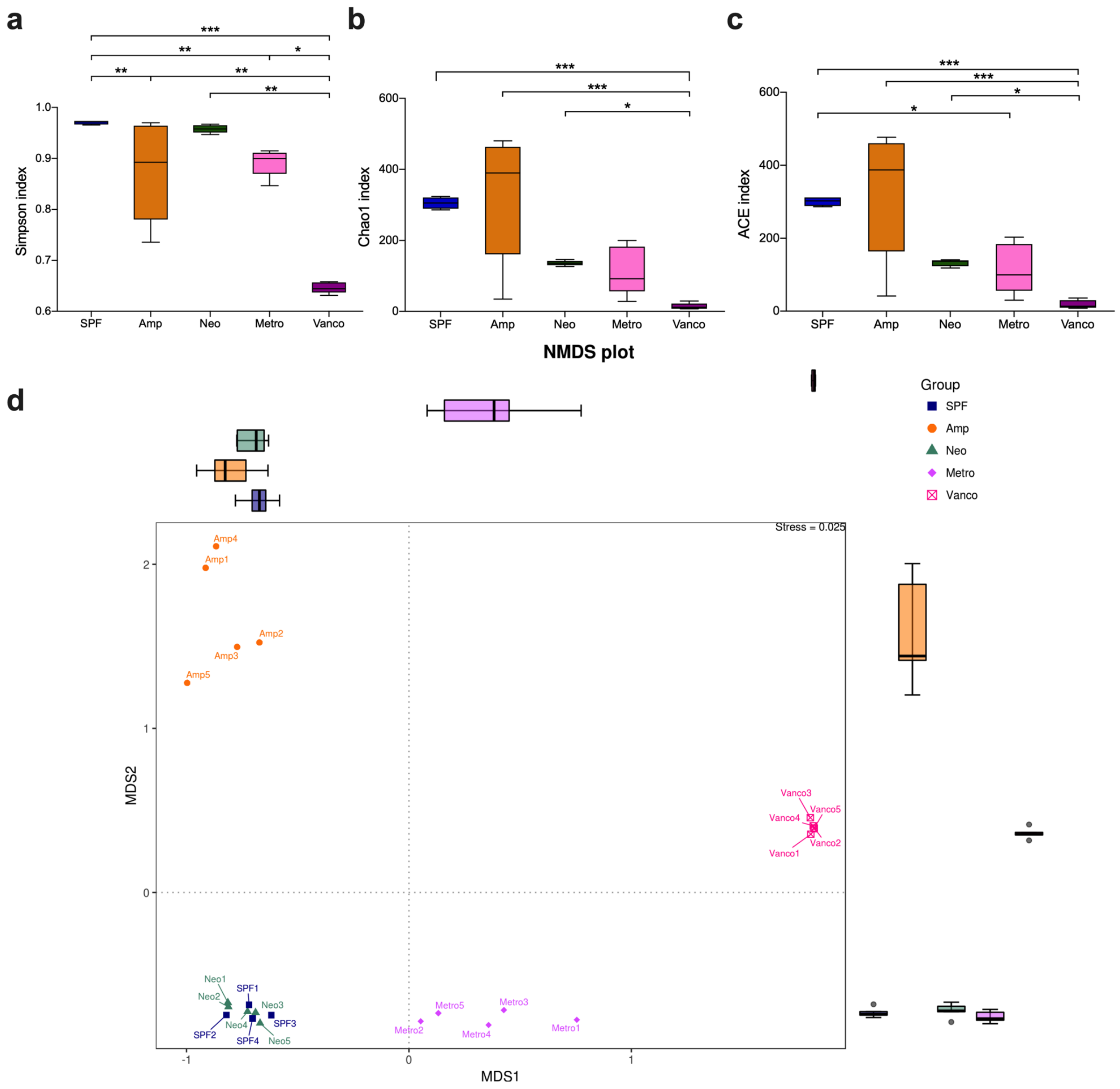

Supplementary Figure 8. Selective antibiotic treatment alters gut microbiome in mice.
(a) Alpha diversity analysis in Simpson index in SPF, Amp-, Neo-, Metro- and Vanco-treated mice (n = 4-5).
(b) Alpha diversity analysis in Chao1 index in SPF, Amp-, Neo-, Metro- and Vanco-treated mice (n = 4-5).
(c) Alpha diversity analysis in ACE index in SPF, Amp-, Neo-, Metro- and Vanco-treated mice (n = 4-5).
(d) Beta diversity analysis in NMDS plot in SPF, Amp-, Neo-, Metro- and Vanco-treated mice (n = 4-5).

Box plots show median (centre line) and interquartile range (IQR) with 1.5 times IQR of the upper and lower quartiles (a, b, c). Data analyzed by Kruskal–Wallis test with LSD post-hoc test (a, b, c). * $P < 0.05$; *** $P < 0.001$.

SPF: specific-pathogen-free; Amp: ampicillin; Neo: neomycin; Metro: metronidazole; Vanco: vancomycin; NMDS: non-metric multidimensional scaling.

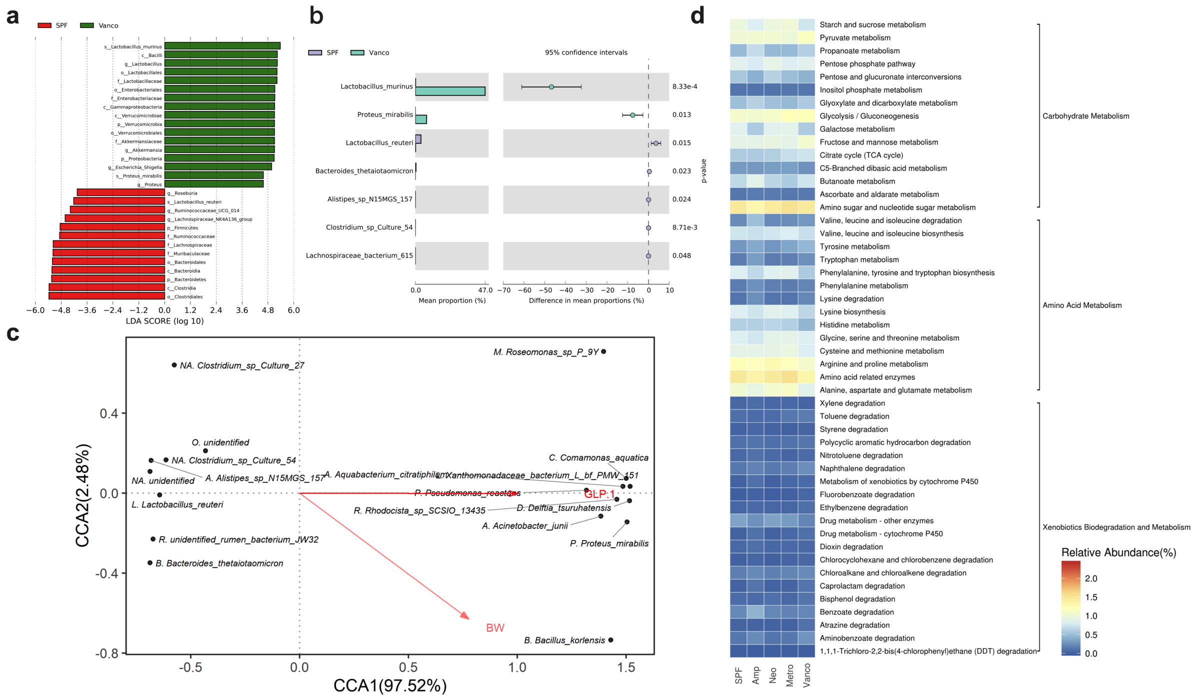

Supplementary Figure 9. Relatively abundance of microbiota at species levels and correlation with GLP-1 levels in selective antibiotic-treated mice.
(a) LEfSe comparison of the bacterial taxa between SPF and Vanco-treated mice (n = 4-5).
(b) STAMP comparison between SPF and Vanco-treated group in species level (n = 4-5).
(c) Canonical correspondence analysis (CCA) shows the correlation between bacteria species and two factors- GLP-1 levels and BW.
(d) Relative abundance heatmap of SPF, Amp-, Neo-, Metro- and Vanco-treated mice via PICRUSt KEGG Level 3 metabolism functional prediction analysis.

Data analyzed by non-parametric factorial Kruskal-Wallis sum-rank test (a), and Welch’s *t*-test (b).

SPF: specific-pathogen-free; Amp: ampicillin; Neo: neomycin; Metro: metronidazole; Vanco: vancomycin; LEfSe: Linear discriminant analysis effect size; STAMP: statistical analysis of metagenomic profiles; CCA: canonical correspondence analysis; PICRUSt: Phylogenetic investigation of communities by reconstruction of unobserved states; KEGG: Kyoto encyclopedia of genes and genomes; BW: body weight.

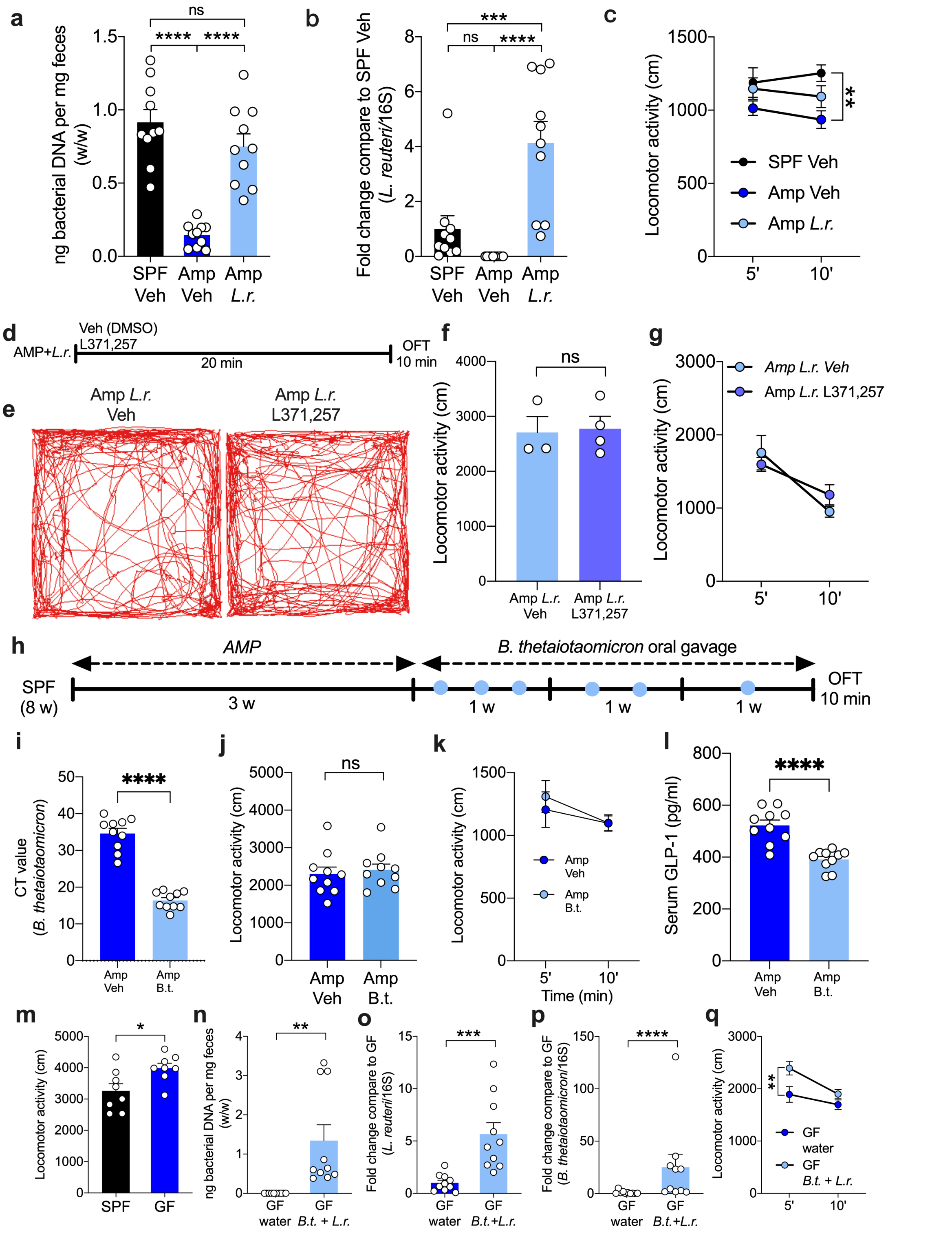

Supplementary Figure 10. *Lactobacillus (L.) reuteri* and *Bacteroides* (*B.*) *thetaiotaomicron* modulate locomotion in ampicillin-treated mice.
(a) Fecal bacterial DNA quantification was detected by Femto DNA quantification methods in SPF+Veh, Amp+Veh, Amp+*L.r.* mice (n=10).

(b) Fecal *L. reuteri* gene expression was normalized to 16S rRNA gene in SPF+Veh, Amp+Veh, Amp+*L.r.* mice. Then compared the fold change to the SPF+Veh group (n=10).
(c) Locomotor activity was tested using the OFT paradigm in 5 minutes bin in SPF+Veh, Amp+Veh, Amp+*L.r.* (n = 10).
(d) Timeline schematic of L-371,257 administration and OFT. The Amp+*L.r.* mice were treated with oxytocin receptor antagonist L-371,257 intranasally 20 minutes before the OFT.
(e) Representative images of trajectories of OFT in Amp+*L.r.*-Veh and Amp+*L.r.*-L371,257 mice.
(f) Locomotor activity was tested using the OFT paradigm in Amp+*L.r.*-Veh and Amp+*L.r.*-L371,257 mice (n = 3-4).
(g) Locomotor activity was tested using the OFT paradigm in 5 minutes bin in Amp+*L.r.*-Veh and Amp+*L.r.*-L371,257 mice (n = 3-4).
(h) Timeline schematic of ampicillin treatment, *B. thetaiotaomicron* colonization, and OFT. Amp mice received 6 times oral gavage in three weeks.
(i) The cycle threshold (CT) value of *B. thetaiotaomicron* in Fecal bacterial DNA in paradigm in Amp+Veh and Amp+*B.t.* mice (n = 10).
(j) Locomotor activity was tested using the OFT paradigm in Amp+Veh and Amp+*B.t.* mice (n = 10).
(k) Locomotor activity was tested using the OFT paradigm in 5 minutes bin in Amp+Veh and Amp+*B.t.* mice (n = 10).
(l) Measurement of serum GLP-1 concentrations in Amp+Veh and Amp+*B.t.* mice (n = 10).
(m) Locomotor activity was tested using the OFT paradigm in SPF (n = 8, data from Supplementary Fig. 1e) and GF mice (n = 8).
(n) Fecal bacterial DNA quantification was detected by Femto DNA quantification methods in GF and GF+*B.r.*+*L.r.* mice (n=10).
(o) Fecal *L. reuteri* gene expression was normalized to 16S rRNA gene in GF and GF+*B.t.*+*L.r.* mice. Then compared the fold change to the GF group. (n=10).
(p) *B. thetaiotaomicron* gene expression was normalized to 16S rRNA gene in GF and GF-*B.t.*+*L.r.* mice. Then compared the fold change to the GF group. (n=10).
(q) Locomotor activity was tested using the OFT paradigm in 5 minutes bin in GF and GF-*B.t.*+*L.r.* mice (n=10).

Data represent mean ± SEM. Data analyzed by one-way ANOVA (a, b), two-way ANOVA with repeated measures (c, g, k, q) with Bonferroni's multiple comparison post-hoc test and two-tailed unpaired *t*-test (f, i, j, l, m, n, o, p). ** $P < 0.01$; *** $P < 0.001$; **** $P < 0.0001$; ns: no significant.

SPF: specific-pathogen-free; Amp: ampicillin; GF: germ-free; Veh: vehicle; *L.r.*: *Lactobacillus reuteri*; *B.t.*: *Bacteroides thetaiotaomicron*; min: minute; w: week.
