# Supplementary material for: The gut microbiota modulate locomotion via vagus-dependent glucagon-like peptide-1 signaling
Source: NPJ Biofilms Microbiomes. 2024 Jan 16;10:2. doi: 10.1038/s41522-024-00477-w (PMC10791613; doi:10.1038/s41522-024-00477-w)
Supplement: Supplementary file 2 — Reporting Summary [file 41522_2024_477_MOESM2_ESM.pdf]

nature portfolio

Corresponding author(s): Wei-Li Wu

Last updated by author(s): Jan 02, 2024

Reporting Summary

Nature Portfolio wishes to improve the reproducibility of the work that we publish. This form provides structure for consistency and transparency in reporting. For further information on Nature Portfolio policies, see our Editorial Policies and the Editorial Policy Checklist.

Statistics

For all statistical analyses, confirm that the following items are present in the figure legend, table legend, main text, or Methods section.

n/a	Confirmed	
☐	☒	The exact sample size (*n*) for each experimental group/condition, given as a discrete number and unit of measurement
☐	☒	A statement on whether measurements were taken from distinct samples or whether the same sample was measured repeatedly
☐	☒	The statistical test(s) used AND whether they are one- or two-sided<br>*Only common tests should be described solely by name; describe more complex techniques in the Methods section.*
☐	☒	A description of all covariates tested
☐	☒	A description of any assumptions or corrections, such as tests of normality and adjustment for multiple comparisons
☐	☒	A full description of the statistical parameters including central tendency (e.g. means) or other basic estimates (e.g. regression coefficient) AND variation (e.g. standard deviation) or associated estimates of uncertainty (e.g. confidence intervals)
☐	☒	For null hypothesis testing, the test statistic (e.g. *F*, *t*, *r*) with confidence intervals, effect sizes, degrees of freedom and *P* value noted<br>*Give P values as exact values whenever suitable.*
☒	☐	For Bayesian analysis, information on the choice of priors and Markov chain Monte Carlo settings
☒	☐	For hierarchical and complex designs, identification of the appropriate level for tests and full reporting of outcomes
☒	☐	Estimates of effect sizes (e.g. Cohen's *d*, Pearson's *r*), indicating how they were calculated

Our web collection on statistics for biologists contains articles on many of the points above.

Software and code

Policy information about availability of computer code

Data collection	Leica Application Suite X version 3.7.2.22383, 3.6.0.20104; Ethovision XT version 14.0.1326, 15.0.1416
Data analysis	Prism version 9.0.0 (121); Ethovision XT version 15.0.1416; ImageJ version 2.14.0/1.54f

For manuscripts utilizing custom algorithms or software that are central to the research but not yet described in published literature, software must be made available to editors and reviewers. We strongly encourage code deposition in a community repository (e.g. GitHub). See the Nature Portfolio guidelines for submitting code & software for further information.

Data

Policy information about availability of data

All manuscripts must include a data availability statement. This statement should provide the following information, where applicable:

- Accession codes, unique identifiers, or web links for publicly available datasets
- A description of any restrictions on data availability
- For clinical datasets or third party data, please ensure that the statement adheres to our policy

The data that support the findings of this study are available from the corresponding author upon reasonable request. Raw data for 16S rRNA gene sequencing and data analysis will be deposited in the Sequence Read Archive (SRA) upon published.

Research involving human participants, their data, or biological material

Policy information about studies with human participants or human data. See also policy information about sex, gender (identity/presentation), and sexual orientation and race, ethnicity and racism.

Reporting on sex and gender	The study did not involve human participants.
Reporting on race, ethnicity, or other socially relevant groupings	The study did not involve human participants.
Population characteristics	The study did not involve human participants.
Recruitment	The study did not involve human participants.
Ethics oversight	The study did not involve human participants.

Note that full information on the approval of the study protocol must also be provided in the manuscript.

Field-specific reporting

Please select the one below that is the best fit for your research. If you are not sure, read the appropriate sections before making your selection.

☒ Life sciences ☐ Behavioural & social sciences ☐ Ecological, evolutionary & environmental sciences

For a reference copy of the document with all sections, see nature.com/documents/nr-reporting-summary-flat.pdf

Life sciences study design

All studies must disclose on these points even when the disclosure is negative.

Sample size	No statistical methods were used to calculate the sample size. Sample size was determined based on prior studies and literature in the field using similar experimental paradigms (Reference: 22, 29, 89, 93, 95)
Data exclusions	In Fig. 3c, 2 ABX-SDV mice were excluded due to weakness or death after surgery. In Fig. 3c. 2 ABX-SDV mice, in Fig. 3f 2 ABX-SDV mice were excluded due to food intake reduction exceeding 30% in comparison to the control group after CCK-8 administration. Otherwise, no data were excluded.
Replication	All data were successfully replicated and data from multiple experiments were pooled at least from two independent trials. For all mouse behavior test, the replication ranged 4-19 mice; sera GLP-1, the replication ranged 7-17 mice; sera CCK, the replication ranged 11-12 mice; IHC, the replication ranged 3-14 mice; 16S rRNA sequence, the replication ranged 4-5 mice; absolute PCR quantification, the replication ranged 5-10 mice; GLP-1+ cells IHC, the replication ranged 2-3 mice.
Randomization	No specific randomization method was used. All mice used were randomly assigned to behavior testing, drug administrations, GLP-1 and CCK measurement, immunofluorescence quantification, subdiaphragmatic vagotomy, focused ultrasound stimulation, 16S rRNA sequence, bacterial colonization, and absolute PCR quantification.
Blinding	Investigators were not blind to treatment groups. The experimenters treating the mice were the same as those analyzing the data. The treatment groups had to be clearly identified throughout the study to prevent cross contamination in the cases of specific-pathogen free, antibiotic-treated and germ-free groups. The sham, SDV, and drug-administered mice had to be consistently identified throughout the study to ensure accuracy. All statistics were performed in an unbiased manner.

Reporting for specific materials, systems and methods

We require information from authors about some types of materials, experimental systems and methods used in many studies. Here, indicate whether each material, system or method listed is relevant to your study. If you are not sure if a list item applies to your research, read the appropriate section before selecting a response.

Materials & experimental systems

n/a	Involved in the study
☐	☒ Antibodies
☒	☐ Eukaryotic cell lines
☒	☐ Palaeontology and archaeology
☐	☒ Animals and other organisms
☒	☐ Clinical data
☒	☐ Dual use research of concern
☒	☐ Plants

Methods

n/a	Involved in the study
☒	☐ ChIP-seq
☒	☐ Flow cytometry
☒	☐ MRI-based neuroimaging

Antibodies

Antibodies used

Primary antibodies and their dilutions were: rabbit anti-c-Fos (1:1000; 2250S; Clone N/A; Cell Signaling Technology), goat anti-choline acetyltransferase (1:1000; AB144P; Clone N/A; Millipore); rabbit anti-Fluorescent Gold (1:1000; AB153-I; Clone N/A; Millipore), and rabbit anti-GLP-1 (1:25; ab22625; Clone N/A; ABcam);. The fluorescence-conjugated secondary antibodies were donkey anti-rabbit (1:500; A-10042; A-31573; ThermoFisher Scientific) and donkey anti-goat (1:500; A-32814; ThermoFisher Scientific)

Validation

Concentration of antibodies were validated based on manufacturing instructions and serial dilutions.
Rabbit anti-c-Fos (1:1000; 2250S; Cell Signaling Technology): https://www.cellsignal.com/products/primary-antibodies/c-fos-9f6-rabbit-mab/2250
Goat anti-choline acetyltransferase (1:1000; AB144P; Millipore): https://www.merckmillipore.com/TW/zh/product/Anti-Choline-Acetyltransferase-Antibody,MM_NF-AB144P
Rabbit anti-Fluorescent Gold (1:1000; AB153-I; Millipore): https://www.merckmillipore.com/TW/zh/product/Anti-Fluorescent-Gold-Antibody,MM_NF-AB153-I
Rabbit anti-GLP-1 (1:25; ab22625; ABcam): https://www.abcam.com/products/primary-antibodies/glp-1-antibody-ab22625.html

Animals and other research organisms

Policy information about studies involving animals; ARRIVE guidelines recommended for reporting animal research, and Sex and Gender in Research

Laboratory animals

Wild-type C57BL/6J (00064) mice used in pre-ABX and post-ABX OFT were obtained through Jackson Laboratory. Wild-type C57BL/6JNarl mice for other experiments were obtained through National Laboratory Animal Center, Taiwan. C57BL/6J germ-free (GF) mice were bred in National Laboratory Animal Center, Taiwan and transfered to Laboratory Animal Center, NCKU. All experiments were performed with male animals. All mice were group-housed in ventilated cage (3-5 mice per cage) with a 13 hr light/ 11hr dark cycle (lights on at 07:00) at 22±1 °C and 55±10% relative humidity. All behaviors test were performed at 8-14 weeks of age.

Wild animals

The study did not use wild animals.

Reporting on sex

All experiments were performed with male animals.

Field-collected samples

The study did not involve field-collected samples

Ethics oversight

All the experimental protocols and the animal care were approved by the National Cheng Kung University (NCKU) Institutional Animal Care and Use Committee (IACUC; #107268 and #108224).

Note that full information on the approval of the study protocol must also be provided in the manuscript.
